# Subtype-Specific Macular Vascular Signatures in Primary Open-Angle, Pseudoexfoliative, and Normal-Tension Glaucoma: OCT Angiography Study

**DOI:** 10.3390/medicina62050941

**Published:** 2026-05-12

**Authors:** Maja L. J. Živković, Marko Zlatanović, Nevena Zlatanović, Mladen Brzaković, Mihailo Jovanović

**Affiliations:** 1 Ophthalmology Clinic, University Clinical Center Niš, Bulevar dr Zorana Đinđića 48, 18000 Nis, Serbia; drzlatanovicmarko@gmail.com; 2 Department of Ophthalmology, Faculty of Medicine, University of Niš, Bulevar dr Zorana Đinđića 81, 18000 Nis, Serbia; 3 Community Health Center Niš in Niš, Vojvode Tankosića 15, 18000 Nis, Serbia; drnevenazlatanovic@gmail.com; 4 Special Hospital for Ophthalmology “Clinic Maja”, Vizantijski Bulevar 8, 18000 Nis, Serbia; brzi.92@hotmail.com; 5 Department of Ophthalmology, Faculty of Medicine, University of Kragujevac, Svetozara Markovića 69, 34000 Kragujevac, Serbia; drmihailojovanovic@gmail.com; 6 Ophthalmology Clinic, University Clinical Center Kragujevac, Zmaj Jovina 30, 34000 Kragujevac, Serbia

**Keywords:** optical coherence tomography angiography, glaucoma subtypes, vessel density, foveal avascular zone, pseudoexfoliative glaucoma, normal-tension glaucoma

## Abstract

*Background and Objectives*: Open-angle glaucoma subtypes share a structural phenotype but differ in pathophysiology: pseudoexfoliative glaucoma (PXG) involves vascular endothelial dysfunction associated with deposition of exfoliative material, whereas normal-tension glaucoma (NTG) reflects primary vascular dysregulation in the absence of elevated intraocular pressure. We characterized subtype-specific OCT angiography (OCTA) profiles obtained from a 3 × 3 mm macular scan and evaluated their discriminatory power for pairwise subtype classification. *Materials and Methods*: This was a single-center, cross-sectional study of 304 eyes: 198 glaucomatous eyes—primary open-angle glaucoma (POAG, glaucoma simplex in our clinical nomenclature), *n* = 102; PXG (glaucoma capsulare), *n* = 68; NTG (glaucoma sine tensio), *n* = 28—and 106 healthy controls. The Cirrus HD-OCT 5000 AngioPlex 3 × 3 mm OCTA protocol was used to assess vessel density (VD), perfusion density, foveal avascular zone (FAZ) morphology, ganglion cell complex (GCC), and retinal nerve fiber layer (RNFL) thickness. Analyses included Kruskal–Wallis tests with Bonferroni post hoc correction, ROC analysis with DeLong comparison of combined versus structural-only models, multivariate regression, and an exploratory XGBoost classifier with SHAP-based interpretation. *Results*: VD Inner and Perfusion Inner were lower in PXG (16.37 ± 3.33%; 0.31 ± 0.05) than in POAG (18.73 ± 3.41%; 0.34 ± 0.05; both *p* < 0.001); Perfusion Inner was also lower than in NTG (*p* < 0.05). FAZ Area was largest in NTG (0.27 ± 0.11 mm^2^) and greater than in PXG (0.19 ± 0.08; *p* < 0.01); FAZ Circularity differed across subtypes (*p* < 0.001). Combined OCTA–structural models outperformed structural-only models for POAG vs. PXG (DeLong *p* = 0.002) and for PXG vs. NTG (AUC = 0.770; *p* = 0.010). Sector-resolved Spearman analysis revealed subtype-specific coupling: in NTG, VD Inner and Perfusion Inner correlated with the inferior RNFL (r = 0.53 and r = 0.52; both *p* < 0.01); in PXG, coupling shifted nasally (r = 0.41 and r = 0.46; both *p* < 0.001). The exploratory XGBoost classifier separated glaucoma from controls with an internal cross-validated AUC of 0.975 ± 0.008 (5-fold CV; not externally validated); FAZ Circularity (mean |SHAP| = 0.418) and FAZ Area (0.411) were the top inter-subtype features, supported by case-level SHAP. RNFL avg and average GCC independently predicted MD across subtypes; in PXG, Perfusion Inner also predicted MD (β = −32.78; *p* = 0.032). *Conclusions*: In this single-center, cross-sectional cohort, OCTA revealed subtype-associated macular microvascular profiles that are complementary to structural OCT. Reduced vessel and perfusion density characterized PXG, whereas FAZ enlargement and reduced circularity distinguished NTG and PXG. Vascular–structural coupling was nasal-predominant in PXG and inferior-predominant in NTG. Combined multimodal models outperformed structural-only approaches. Macular perfusion additionally predicted MD in PXG. The XGBoost/SHAP analysis is exploratory; prospective and externally validated studies are required before clinical deployment.

## 1. Introduction

Glaucoma is a heterogeneous group of progressive optic neuropathies that share a common final pathway: irreversible loss of retinal ganglion cells (RGCs), characteristic cupping of the optic nerve head, and corresponding visual field defects [1]. It is the leading cause of irreversible blindness worldwide, accounting for an estimated 8% of global blindness and 3.2 million blind individuals as of 2020 [2]. Widely cited epidemiological projections estimate that 76 million people were living with glaucoma in 2020, with this figure projected to rise to 111.8 million by 2040, driven predominantly by aging populations in Asia and Africa [3]. The disease burden is compounded by its silent natural history: the most prevalent forms of open-angle glaucoma—primary open-angle glaucoma (POAG), pseudoexfoliative glaucoma (PXG), and normal-tension glaucoma (NTG)—are clinically asymptomatic in early stages, and the majority of cases remain undiagnosed until significant, irreversible structural and functional damage has already occurred [1,4].

Despite substantial advances in imaging and perimetry, the central challenge in glaucoma management has shifted from merely identifying the disease to characterizing it by subtype. POAG, PXG, and NTG share a common structural phenotype—optic nerve cupping, RNFL thinning, and corresponding visual field loss—yet differ in pathophysiology, the relative contribution of intraocular pressure (IOP)-dependent versus IOP-independent injury mechanisms, and consequently, in therapeutic and monitoring implications [5]. The central question this study addresses is whether optical coherence tomography angiography (OCTA) provides diagnostically informative, subtype-associated microvascular signals that go beyond what is currently accessible through standard structural OCT and automated perimetry alone.

POAG (glaucoma simplex) is the most prevalent subtype, accounting for approximately 74% of all glaucoma cases globally [3]. PXG (glaucoma capsulare) is the most common identifiable cause of secondary open-angle glaucoma worldwide, potentially affecting up to 70 million individuals [6]. It is characterized by progressive accumulation of fibrillar pseudoexfoliative material in the anterior segment, zonular fibers, trabecular meshwork, and the walls of ocular and systemic blood vessels. The systemic vascular component of pseudoexfoliative disease—increased plasma endothelin-1, reduced nitric oxide bioavailability, elevated plasma homocysteine, and increased aortic stiffness—contributes to vascular endothelial injury that operates independently of IOP elevation [7,8]. Bourouki et al. provided direct mechanistic support for this vascular phenotype by demonstrating measurable endothelial dysfunction and arterial stiffness in PXG patients, associated with elevated circulating apoptotic endothelial microparticles [9]. NTG (glaucoma sine tensio) is defined as glaucomatous optic neuropathy with corresponding visual field defects in the context of IOP consistently at or below 21 mmHg [10,11]; it i s particularly prevalent in Asian populations and accounts for 20–40% of POAG diagnoses in European cohorts [12]. NTG eyes show larger, deeper cupping, more frequent disc hemorrhages, and more paracentral visual field defects than POAG eyes at equivalent structural severity—features consistent with a prominent vascular contribution to optic nerve injury [11,13]. Although IOP reduction remains the only evidence-based intervention, it is insufficient to halt progression in a substantial minority of NTG patients, implicating IOP-independent mechanisms, including impaired autoregulation of ocular blood flow, primary vascular dysregulation (Flammer syndrome), and vasospasm [12,14]. The vascular hypothesis predicts that perifoveal capillary dropout and FAZ enlargement should be detectable by OCTA as an early manifestation of primary vascular pathology [15].

OCTA is a functional extension of structural OCT that enables non-invasive, depth-resolved imaging of the retinal and choroidal microvasculature without intravenous dye injection [16]. Layer-specific visualization of the superficial capillary plexus, deep capillary plexus, outer retina, and choriocapillaris is obtained in a single scanning session, providing simultaneous structural and vascular information. The reduction in macular and peripapillary vessel density in glaucoma compared with healthy eyes is now well established [17], and macular GCC thickness has been identified as the optimal structural measure for detecting macular progression [18]. However, most published studies have analyzed glaucoma as a single diagnostic category—predominantly POAG versus healthy controls—with limited attention to subtype-specific OCTA profiles [17,18]. The clinically decisive question in daily practice is not glaucoma versus healthy, which structural OCT already addresses well, but rather identifying which subtype a given patient has, as this determines the dominant pathogenic mechanism, the expected rate of progression, and the optimal therapeutic and monitoring strategy. A small number of studies have directly compared OCTA parameters between POAG and PXG, consistently reporting lower peripapillary and macular vessel density in PXG at comparable structural severity [6,19]. For NTG, early reports have supported the hypothesis that FAZ enlargement and perifoveal capillary dropout represent a primary vascular manifestation [12,15]. A direct three-way comparison of macular OCTA profiles across POAG, PXG, and NTG within a single study population, with consistent methodology and including both subtype discrimination and structure–function regression analyses augmented by machine-learning feature importance, has not been published to date. The present study addresses this gap.

This cross-sectional observational study was designed around four primary research aims and one exploratory aim. The first aim was to characterize macular OCTA profiles—vessel density (central, inner, full), perfusion density (central, inner, full), and FAZ morphology (area, perimeter, circularity)—across POAG, PXG, NTG, and a healthy control group, identifying statistically significant between-group differences that may reflect subtype-associated vascular pathology. The second aim was to evaluate the discriminative power of individual macular OCTA parameters and their combination with structural OCT parameters (GCC, RNFL) using ROC analysis with DeLong pairwise comparisons. The third aim was to examine structural–vascular correlations within each subtype and determine whether vascular–structural dissociation can be identified as a feature of any particular subtype. The fourth aim was to determine, via multivariate linear regression, whether macular OCTA parameters independently predict visual field mean deviation (MD) after controlling for structural OCT parameters, age, and sex. The exploratory fifth aim was to apply an XGBoost classifier with SHAP-based feature importance to provide an interpretable, data-driven ranking of parameter relevance for glaucoma versus control discrimination and inter-subtype classification; this component is reported as exploratory and is not externally validated.

## 2. Materials and Methods

### 2.1. Study Design and Participants

All participants were recruited from the Special Hospital for Ophthalmology “Clinic Maja” in Niš, Serbia, between October 2018 and October 2021. This single-center, case–control, cross-sectional study was approved by the Ethics Committee of the Special Hospital for Ophthalmology “Clinic Maja” on 25 September 2018 (approval number 09/09-2018) and was conducted in accordance with the Declaration of Helsinki. Written informed consent was obtained from all participants. The single-center cross-sectional design and its resulting limitations on causal inference and generalizability are explicitly addressed in the Limitations subsection of the Discussion.

A total of 304 eyes were included: 198 glaucomatous eyes (POAG, *n* = 102; PXG, *n* = 68; NTG, *n* = 28) and 106 healthy control eyes. The vast majority of glaucomatous eyes were from distinct individuals; based on matching age, sex, and laterality across adjacent records in the source data, only six pairs of glaucomatous eyes (representing approximately 3% of the cohort) were from the same patient. Statistical analyses were therefore performed at the eye level, and a sensitivity analysis restricted to one eye per patient (the eye with the worst mean deviation when both eyes were eligible) was carried out as a robustness check (Section 2.5). All glaucoma patients were receiving established topical antiglaucoma therapy at the time of enrollment. Each participant underwent a complete ophthalmic examination, including best-corrected visual acuity (BCVA), slit-lamp biomicroscopy, Goldmann applanation tonometry, gonioscopy, dilated fundus examination, standard automated perimetry, structural OCT, and OCTA.

Inclusion criteria for glaucoma patients: confirmed diagnosis of POAG, PXG, or NTG; age 40–80 years; BCVA ≥ 0.3 (Snellen decimal); refractive error within ±5.0 diopters sphere and ±2.5 diopters cylinder; and reliable visual field results (fixation loss ≤20%, false-positive and false-negative errors ≤15%). A glaucoma diagnosis required a reproducible glaucomatous visual field defect on two consecutive tests and corresponding structural changes on examination. The clinical subtype assignment (POAG/PXG/NTG) was made by the attending ophthalmologist based on standard diagnostic criteria, including IOP history, gonioscopy, slit-lamp evidence of pseudoexfoliative material, and the structural and functional pattern of optic neuropathy.

*Note on nomenclature.* Throughout this manuscript, the international abbreviations POAG, PXG, and NTG serve as primary identifiers for the three open-angle glaucoma subtypes. The corresponding classical Latin clinical descriptors—glaucoma simplex, glaucoma capsulare, and glaucoma sine tensio—are retained only as parenthetical equivalents at first mention in the Abstract and Introduction, reflecting the clinical nomenclature used at our institution.

Inclusion criteria for controls: normal IOP (<21 mmHg); BCVA >0.8; normal optic nerve head appearance; normal visual field; no family history of glaucoma; and absence of systemic or ocular disease known to affect retinal vasculature. Controls were screened to exclude diabetes mellitus, uncontrolled systemic hypertension, prior cerebrovascular events, and any cardiovascular condition with documented systemic vascular involvement. Visual field testing was performed in all participants, including healthy controls, to confirm normal perimetric indices in the reference group.

Exclusion criteria (both groups): media opacity precluding reliable OCT or OCTA imaging (signal strength < 7/10); history of intraocular surgery other than uncomplicated phacoemulsification; diabetic retinopathy; retinal vascular occlusion; age-related macular degeneration; other optic neuropathies; systemic diseases with known ocular vascular involvement; and use of medications affecting retinal vasculature.

*Topical antiglaucoma medication.* All glaucoma patients in this cohort were receiving topical antiglaucoma therapy at the time of OCTA imaging, consistent with the institutional standard of care. In our practice, prostaglandin analogs are the dominant first-line agents, with fixed-combination preparations (typically prostaglandin analogue with timolol, or dorzolamide–timolol) reserved for patients not at target IOP on monotherapy. Detailed numerical drug-class data, daily dose, and duration of therapy were not available in a structured format across the entire analytical dataset and were therefore not included as covariates in the regression or machine-learning analyses.

### 2.2. Visual Field Testing

Standard automated perimetry was performed with the Humphrey Field Analyzer II (Carl Zeiss Meditec, Dublin, CA, USA) using the 24-2 SITA Standard protocol. The primary functional outcome was mean deviation (MD, dB); pattern standard deviation (PSD, dB) served as a secondary measure of focal defect severity. Glaucoma severity was classified according to the Hodapp–Parrish–Anderson scale: early (MD > −6 dB), moderate (−12 dB ≤ MD ≤ −6 dB), and severe (MD < −12 dB). Visual field testing was performed in all participants, including healthy controls, to confirm normal perimetric indices in the reference group.

### 2.3. Structural OCT: RNFL and GCL + IPL Assessment

Structural imaging was performed using the Cirrus HD-OCT Model 5000 (Carl Zeiss Meditec, Dublin, CA, USA), a spectral-domain OCT system with axial resolution of 5 µm, lateral resolution of 15 µm, a scan rate of 27,000 A-scans per second, and a central wavelength of 840 nm. Imaging was performed with a dilated pupil; only scans with signal strength ≥ 6/10 were included.

Peripapillary RNFL thickness was measured using the Optic Disc Cube 200 × 200 protocol, which acquires a 6 × 6 mm data cube centered on the optic nerve head. Global average RNFL thickness and four-quadrant measurements (superior, nasal, inferior, temporal) were extracted for analysis. The instrument automatically identifies the optic disc boundary and computes the average circumpapillary RNFL thickness along a 3.46 mm diameter circle. The Cirrus AutoCenter™ function was used to ensure reproducible scan centration.

Macular ganglion cell–inner plexiform layer (GCL + IPL) thickness was measured using the Macular Cube 512 × 128 protocol with the Ganglion Cell Analysis (GCA) algorithm, which segments and measures GCL + IPL thickness within an elliptical annulus (2.0 mm vertical radius, 2.4 mm horizontal radius) centered on the fovea. The GCA algorithm isolates GCL + IPL from the RNFL, avoiding the diagnostic inflation associated with RNFL-inclusive GCC measurements on other platforms [20]. Six sectoral measurements were obtained: superonasal (GC1), superior (GC2), superotemporal (GC3), inferotemporal (GC4), inferior (GC5), and inferonasal (GC6), along with global average and minimum GCL + IPL thickness. Average and minimum GCL + IPL thickness, together with the average RNFL, formed the principal structural feature set used in subsequent analyses [20].

### 2.4. OCT Angiography: Macular 3 × 3 mm Protocol and Parameters

OCTA was performed using the Cirrus HD-OCT 5000 with AngioPlex (Carl Zeiss Meditec, Dublin, CA, USA). All OCTA imaging used a standardized 3 × 3 mm macular scan protocol centered on the fovea. The superficial capillary plexus slab was defined as extending from 3 µm below the internal limiting membrane to 15 µm below the inner plexiform layer. Scans with signal strength < 7/10 or motion artifacts obscuring >10% of the imaging area were rejected and repeated. The following parameters were quantified from the 3 × 3 mm macular OCTA scan: vessel density (VD Central, VD Inner, VD Full; %); perfusion density (Perf Central, Perf Inner, Perf Full); and FAZ morphology (FAZ Area in mm^2^, FAZ Perimeter in mm, FAZ Circularity Index).

### 2.5. Statistical Analysis

All statistical analyses were conducted using IBM SPSS Statistics, Version 22.0 (IBM Corp., Armonk, NY, USA), and Python 3.12 with the scipy, scikit-learn, XGBoost, and SHAP libraries. A two-tailed *p*-value < 0.05 was considered statistically significant.

Descriptive statistics are reported as mean ± standard deviation (SD) for continuous variables and as counts and percentages for categorical variables. Normality of continuous variables was assessed using the Shapiro–Wilk test. Because OCTA parameters were not normally distributed, nonparametric methods were used as the primary framework for group comparisons. Differences across the three glaucoma subtypes were assessed using the Kruskal–Wallis H test, with post hoc pairwise comparisons using the Mann–Whitney U test with Bonferroni correction for three pairwise comparisons per parameter. Spearman rank-order correlations (ρ) were computed for bivariate associations between OCTA and structural parameters, both in the combined glaucoma cohort and separately by subtype. In addition to the primary OCTA–structural pairs, a sector-resolved correlation matrix covering all nine macular OCTA parameters against all seven structural parameters (Avg GCC, Min GCC, four RNFL sectoral thicknesses, plus RNFL average) was computed separately by subtype to characterize topographic patterns of vascular–structural coupling.

Approach to multiplicity. The analyses presented here are organized around four pre-specified research aims and one exploratory aim, rather than as parallel, independent hypothesis tests. Within each aim, a coherent approach to controlling type I error was applied: pairwise post hoc Mann–Whitney comparisons after Kruskal–Wallis used Bonferroni correction across the three subtype pairs (×3); pairwise model comparisons in the ROC analysis used DeLong testing against a structural-only reference; regression models within each subtype used age and sex as covariates and a single primary OCTA predictor per model; and the machine-learning component is reported throughout as exploratory. Cross-aim convergence—between Kruskal–Wallis, ROC, regression, and SHAP-based feature importance—is therefore presented as triangulated evidence rather than as evidence from independent statistical tests. We do not claim a global family-wise error rate across all aims, nor do we adjust for one. Findings that depend on a single aim or on small subgroups are flagged as exploratory in the Results.

Approach to inter-eye correlation. Because the vast majority of patients in this cohort contributed only one eye to the analytical dataset—with only six pairs of glaucomatous eyes (≈3% of the glaucoma cohort) representing bilateral inclusion of the same patient—the expected impact of intra-cluster correlation between fellow eyes on the analyses is small. The primary analyses were therefore performed at the eye level. As a pre-specified robustness check, the principal Kruskal–Wallis comparisons, the pairwise ROC analyses (structural-only, OCTA-only, and combined logistic-regression models), and the multivariate regression models were repeated on a one-eye-per-patient subset, retaining the eye with the worse mean deviation when both eyes were eligible. Concordance between the full and one-eye analyses is reported in the Results.

Receiver operating characteristic (ROC) analysis was performed for pairwise subtype discrimination. For each binary comparison, AUCs were calculated for each individual OCTA and structural parameter (17 variables in total), and 95% confidence intervals were estimated using the DeLong method. Combination models (structural-only, OCTA-only, and combined) were derived from 5-fold cross-validated logistic regression with L2 regularization (C = 0.1) applied to standardized predictors. DeLong’s test was used to compare the OCTA-only and combined models against the structural-only reference. Youden’s index was used to identify the optimal sensitivity–specificity operating point on each ROC curve.

Multivariate linear (OLS) regression was performed with visual field MD (dB) as the dependent variable. Structural parameters (RNFL avg, Avg GCC) and OCTA parameters (VD Inner, Perf Inner, FAZ Area) were entered separately as primary predictors in individual models, each adjusted for age and sex as covariates. Model output is reported as regression coefficients (β) with standard errors and 95% confidence intervals, along with model-level diagnostics (F-statistic with *p*-value, coefficient of determination R^2^, adjusted R^2^, residual standard error). The incremental contribution of each predictor beyond age and sex was summarized as the difference in adjusted R^2^ between the full model and a covariate-only reference model. Because the NTG subgroup contained only 28 eyes, all regression results within this subgroup are presented as exploratory and should be interpreted with caution.

As an exploratory aim, an XGBoost gradient-boosting classifier (n_estimators = 200, max_depth = 4, learning_rate = 0.05) was trained with 5-fold stratified cross-validation for two tasks: (1) binary classification of glaucoma versus healthy controls; and (2) multiclass classification of the three glaucoma subtypes. Performance metrics for this classifier reflect internal cross-validation only; no held-out external test set was available, and the AUC of 0.975 ± 0.008 reported for the glaucoma-vs-control task should therefore be regarded as an internal performance estimate that may overstate performance in independent populations. Feature importance was quantified using SHAP (SHapley Additive exPlanations) values, providing an interpretable, model-agnostic decomposition of each feature’s contribution to model predictions. Two complementary SHAP analyses were performed: (i) cohort-level (global) importance, summarized as the mean absolute SHAP value per feature across all patients; and (ii) case-level (local) importance, obtained by extracting per-sample SHAP values for representative individual patients (one per subtype, selected as the case with the highest model probability for the correct class), used to verify that the global feature ranking reflected genuine per-patient contributions rather than artifacts of averaging.

Extended analytical outputs that complement the main text tables and figures—including the complete sector-resolved Spearman correlation matrix, full ROC statistics for each parameter across all pairwise comparisons, detailed regression diagnostics with coefficients and confidence intervals for all covariates, and the case-level SHAP analysis with per-patient ROC curves for the top-performing individual parameters—are available in the Supplementary Materials.

## 3. Results

A total of 304 eyes were included: 198 from glaucoma patients and 106 from healthy controls. The glaucoma cohort comprised three subtypes: POAG (*n* = 102), PXG (*n* = 68), and NTG (*n* = 28). Baseline characteristics are presented in Table 1. Age and sex distributions were broadly comparable across the four groups: the Kruskal–Wallis test did not reveal a significant overall difference in age (mean values 64.4–66.7 years across groups; Kruskal–Wallis *p* > 0.05), and female proportion ranged from 54.9% to 60.7%. MD values were negative across all glaucoma subtypes, with the most severe deficit in PXG (MD −6.6 ± 6.9 dB). PSD was highest in NTG (7.27 ± 1.54 dB), significantly exceeding the other two subtypes (*p* < 0.001), reflecting the more localized pattern of visual field loss in normal-tension glaucoma.

### 3.1. Between-Subtype Differences in Macular OCTA and Structural Parameters

**Figure 1:**
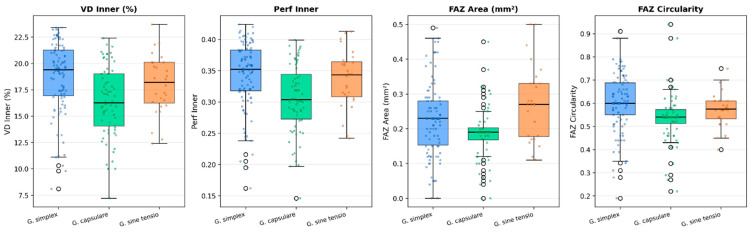
Box plots of the top discriminating OCT parameters across glaucoma subtypes, with jittered individual data points overlaid. Boxes show interquartile range; horizontal line = median. POAG (blue), PXG (green), NTG (orange). VD Inner and Perfusion Inner are lowest in PXG; FAZ Area and FAZ Circularity show distinct patterns across subtypes.

Kruskal–Wallis tests revealed statistically significant differences among the three glaucoma subtypes for all macular OCTA parameters (vessel density, perfusion density, FAZ morphology) and for selected structural parameters. After Bonferroni correction for the three pairwise comparisons per parameter, central subfield thickness (CST), Min GCC, and the nasal, inferior, and temporal RNFL sectors did not differ significantly across subtypes. Full results are presented in Table 2. Box plots of the top discriminating parameters are shown in Figure 1.

The most pronounced inter-subtype differences in macular OCTA were observed in VD Inner and Perfusion Inner. PXG showed the lowest values (VD Inner 16.37 ± 3.33%; Perfusion Inner 0.31 ± 0.05), significantly lower than those in POAG (VD Inner 18.73 ± 3.41%, *p* < 0.001; Perfusion Inner 0.34 ± 0.05, *p* < 0.001). NTG was intermediate (VD Inner 17.99 ± 2.79%; Perfusion Inner 0.34 ± 0.05), with Perfusion Inner significantly higher than in PXG (*p* < 0.05 after Bonferroni correction). VD Full and Perfusion Full showed the same pattern. These findings indicate that pseudoexfoliative glaucoma is associated with greater macular vascular impairment than both primary open-angle and normal-tension glaucoma in this cohort.

FAZ morphology showed consistent differences across all three parameters. NTG exhibited the largest FAZ Area (0.27 ± 0.11 mm^2^), significantly greater than in PXG (0.19 ± 0.08 mm^2^, *p* < 0.01); POAG was also larger than PXG (0.23 ± 0.11 mm^2^, *p* < 0.01). FAZ Circularity was significantly lower in PXG (0.54 ± 0.11) than in POAG (0.60 ± 0.12, *p* < 0.001), indicating greater FAZ shape irregularity in pseudoexfoliative disease. The enlarged FAZ in NTG is consistent with preferential foveal microvascular disruption in normal-tension glaucoma.

Structural parameters showed the most severe damage in PXG. Avg GCC was significantly lower in PXG (71.13 ± 12.76 μm) than in NTG (77.68 ± 9.85 μm, *p* < 0.05). RNFL Superior was selectively reduced in PXG relative to POAG (89.06 ± 25.92 vs. 98.73 ± 19.68 μm, *p* < 0.05) and NTG (104.00 ± 17.92 μm, *p* < 0.05), consistent with the predilection of pseudoexfoliative glaucoma for superior arcuate defects. CST did not differ across groups (*p* = 0.866).

#### Sensitivity Analysis: One Eye per Patient

To evaluate whether the small fraction of bilateral inclusion materially influenced the principal findings, all primary statistical comparisons were repeated on a one-eye-per-patient subset of 191 glaucomatous eyes (POAG, *n* = 98; PXG, *n* = 65; NTG, *n* = 28) constructed by retaining the eye with the worse mean deviation per patient. Concordance with the full-cohort analyses was high. Kruskal–Wallis comparisons of all nine macular OCTA parameters retained the same significance pattern (all eight parameters significant at *p* < 0.05 in both analyses; VD Inner full-cohort *p* < 0.001 vs. one-eye *p* < 0.001; Perfusion Inner full-cohort *p* < 0.001 vs. one-eye *p* < 0.001; FAZ Area full-cohort *p* < 0.001 vs. one-eye *p* < 0.001; FAZ Circularity full-cohort *p* < 0.001 vs. one-eye *p* < 0.001). Bonferroni-corrected pairwise post hoc tests yielded an identical sequence of significant subtype contrasts in both analyses. Cross-validated ROC discrimination was concordant across the three pairwise comparisons (POAG vs. PXG OCTA-only AUC 0.715 full vs. 0.702 one-eye; POAG vs. PXG combined AUC 0.690 vs. 0.677; PXG vs. NTG OCTA-only AUC 0.763 vs. 0.753; PXG vs. NTG combined AUC 0.743 vs. 0.781; POAG vs. NTG combined AUC 0.673 vs. 0.636). The regression of macular Perfusion Inner on MD in PXG retained statistical significance with virtually unchanged effect size (full β = −32.86, *p* = 0.035; one-eye β = −32.86, *p* = 0.038), and RNFL avg remained the most consistent structural predictor of MD across subtypes in both analyses. Detailed comparison of full-cohort and one-eye-per-patient statistics is provided in Supplementary Table S4.

### 3.2. Structural–Vascular Correlations by Subtype

**Figure 2:**
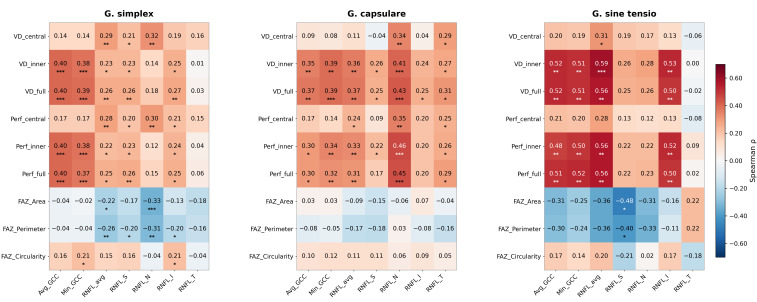
Spearman correlation heatmap: macular OCTA vs. structural parameters by glaucoma subtype. Cell annotations show r value and significance (* *p* < 0.05, ** *p* < 0.01, *** *p* < 0.001). Color scale: blue = positive, red = negative correlation.

Spearman correlations between the principal macular OCTA parameters (VD Inner, Perf Inner, FAZ Area, FAZ Perimeter) and key structural parameters (Avg GCC, RNFL avg) are summarized in Table 3, with subtype-stratified heatmaps shown in Figure 2. The complete correlation matrix, covering all nine macular OCTA parameters against all seven structural parameters, is provided in Table S2 of the Supplementary Materials, allowing inspection of sector-specific RNFL associations (Superior, Nasal, Inferior, Temporal) and Min GCC relationships, which are consolidated in the main table.

In the combined cohort (*n* = 198), VD Inner and Perfusion Inner showed statistically significant moderate correlations with Avg GCC (r = 0.41 and r = 0.40, both *p* < 0.001) and with RNFL avg (r = 0.34 and r = 0.32, both *p* < 0.001), confirming partial structural–vascular coupling with substantial residual independence. FAZ Area showed no significant correlation with Avg GCC in any subtype (all *p* > 0.10), indicating that FAZ morphology captures structurally independent information.

Subtype-stratified analysis revealed clinically relevant heterogeneity. In NTG, VD Inner showed the strongest correlations with structural parameters of any subtype (vs. Avg GCC: r = 0.52, *p* = 0.005; vs. RNFL avg: r = 0.59, *p* < 0.001), a pattern consistent with shared microvascular involvement in normal-tension glaucoma. Effect sizes were smaller in PXG (r = 0.35–0.36), a finding compatible with an additional mechanical component of optic nerve damage in pseudoexfoliative disease. The complete correlation matrix in Table S2 (Supplementary Materials) further shows that in NTG, VD Inner and Perfusion Inner correlate strongly with the RNFL inferior sector specifically (r = 0.53 and r = 0.52, respectively; both *p* < 0.01), while in PXG the strongest sectoral coupling is with the RNFL nasal sector (r = 0.41 and r = 0.46; both *p* < 0.001)—consistent with subtype-associated topographic patterns of vascular–structural coupling that the consolidated main table does not resolve.

### 3.3. Diagnostic ROC Analysis

**Figure 3:**
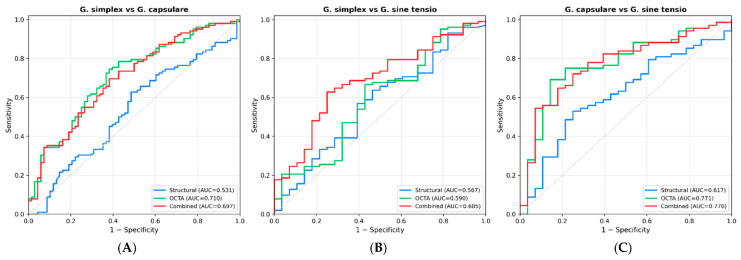
ROC curves for structural-only, OCTA-only, and combined models across the three pairwise glaucoma subtype comparisons. (**A**) POAG vs. PXG: OCTA-only model achieves AUC = 0.695, outperforming structural-only (AUC = 0.531). (**B**) POAG vs. NTG: combined model (AUC = 0.667) outperforms structural-only (AUC = 0.567). (**C**) PXG vs. NTG: OCTA-only (AUC = 0.765) and combined (AUC = 0.770) both outperform the structural reference (AUC = 0.617).

ROC analysis was performed for all pairwise comparisons between the three glaucoma subtypes. The best-performing individual parameters and the combined, OCTA-only, and structural-only multivariable models are presented in Table 4, with ROC curves for the three main models shown in Figure 3. Complete ROC statistics for every individual parameter (all 17 OCTA and structural variables, including 95% CIs by the DeLong method, Youden-optimal sensitivity and specificity) across all three pairwise comparisons are provided in Table S1 of the Supplementary Materials, and the corresponding ROC curves for the top five individual parameters per comparison are shown in Figure S2.

For POAG vs. PXG, Perf Inner, VD Inner, and FAZ Circularity achieved nearly identical top individual AUCs (0.706, 0.705, and 0.703, respectively; see Table S1 for complete per-parameter 95% CIs, sensitivities, and specificities). All three OCTA parameters substantially outperformed structural parameters (RNFL avg AUC = 0.597; Avg GCC AUC = 0.548), and the structural-only model performed near chance (AUC = 0.531). The OCTA-only model achieved an AUC of 0.695 (DeLong *p* = 0.002 vs. structural). The absolute differences in AUC between the OCTA-only (0.695) and combined (0.684) models are small and clinically modest in isolation, despite reaching statistical significance against the near-chance structural reference. The comparison demonstrates added value over structural OCT for this subtype pair, but does not establish that any single model has stand-alone diagnostic accuracy in the clinically useful range. Table S2A illustrates the three near-equivalent top individual OCTA parameters—a finding that argues against relying on any single OCTA biomarker for this comparison and supports multivariable combination as a strategy worth prospective validation.

For POAG vs. NTG, no individual parameter exceeded an AUC of 0.62 (Table S1), reflecting the structurally and vascularly similar phenotypes of these two subtypes at the macular level. Structural parameters performed near chance (structural-only AUC = 0.567). The OCTA-only model achieved an AUC of 0.563 (DeLong *p* = 0.967), and the combined model reached an AUC of 0.667 (DeLong *p* = 0.171 vs. structural). These results indicate that no simple classifier confidently discriminates POAG from NTG using macular OCTA and structural parameters alone. FAZ morphology captures the subtype-associated vascular signal of NTG but only with modest magnitude in the absence of complementary peripapillary information.

For PXG vs. NTG, FAZ Area achieved the highest individual OCTA AUC (0.701), followed by FAZ Perimeter (0.670), with structural parameters trailing (Avg GCC AUC = 0.659; RNFL avg AUC = 0.651; Table S1, Figure S2C). The OCTA-only model achieved an AUC of 0.765, and the combined model an AUC of 0.770, with the latter significantly outperforming the structural-only reference (DeLong *p* = 0.010). This performance reflects the contrasting vascular profiles of the two subtypes: PXG with predominantly macular vascular and perfusion compromise, and NTG with primary foveal microvascular disruption, expressed as an enlarged, geometrically distorted FAZ.

### 3.4. Structure–Function Regression

Multivariate linear regression was used to identify independent predictors of MD within each subtype, with age and sex as covariates. The main regression coefficients and *p*-values are summarized in Table 5. Complete model diagnostics—including coefficients and 95% CIs for all covariates (intercept, age, sex, parameter of interest), F-statistics with *p*-values, R^2^ and adjusted R^2^, and residual standard errors—are provided in Table S3 of the Supplementary Materials.

In POAG (*n* = 102), average RNFL was the strongest predictor (β = 0.128, *p* = 0.002); average GCC approached significance (β = 0.082, *p* = 0.064). The full model (Table S3) explained 12.4% of the variance in MD (adjusted R^2^ = 0.097; F = 4.62, *p* = 0.005), and neither age nor sex reached statistical significance independently.

In PXG (*n* = 68), both structural predictors were substantially stronger than in POAG: RNFL avg (β = 0.154, *p* = 0.004) and Avg GCC (β = 0.189, *p* = 0.004), consistent with tight structure–function coupling in pseudoexfoliative glaucoma. Notably, Perfusion Inner emerged as a significant independent predictor in this subtype (β = −32.783, 95% CI −62.75 to −2.82, *p* = 0.032). The full model explained 28.0% of the variance in MD (adjusted R^2^ = 0.190; F = 3.11, *p* = 0.045; Table S3), indicating that macular perfusion captures additional predictive information for visual field loss specifically in pseudoexfoliative disease, beyond what is explained by structural parameters alone.

In NTG (*n* = 28; exploratory), RNFL avg remained a significant predictor (β = 0.190, *p* = 0.031). Perfusion Inner approached significance (β = 35.732, *p* = 0.083; Table S3) in a positive direction—opposite to that observed in PXG—but the limited sample size precludes firm inference, so this finding is reported as exploratory.

Across the three subtypes, structural parameters (RNFL, GCC) were the dominant and most consistent predictors of MD, whereas macular OCTA parameters were not independent predictors after adjustment for age and sex in POAG or NTG. This dissociation is interpretable: vascular parameters appear to capture a dimension of glaucomatous injury that is mechanistically distinct from and not directly upstream of current perimetric functional loss in these two subtypes. The exception in PXG, in which Perfusion Inner reached significance, is consistent with the dual pathogenic burden of pseudoexfoliative disease, in which vascular endothelial dysfunction has been described as contributing to neuroretinal damage alongside mechanical trabecular obstruction.

### 3.5. XGBoost Classification and SHAP Feature Importance

An exploratory XGBoost classifier was trained to distinguish all glaucoma from controls and achieved a 5-fold cross-validated AUC of 0.975 ± 0.008. As noted in the Methods, this performance reflects internal cross-validation only and is therefore likely to be optimistic relative to performance in independent populations; external validation is required before any clinical interpretation. For glaucoma subtype classification (3-class: POAG/PXG/NTG), 5-fold cross-validated accuracy was 0.667 ± 0.054.

**Figure 4:**
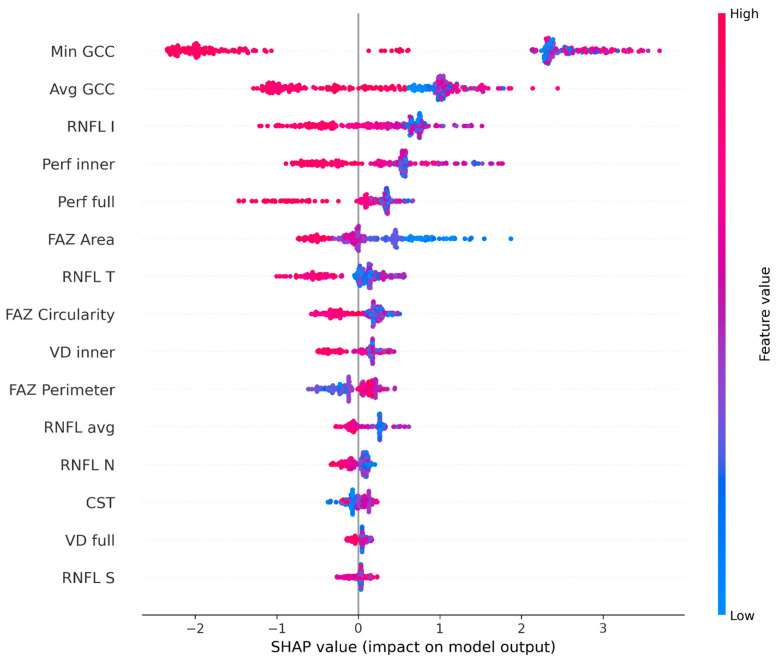
SHAP summary plot (beeswarm): XGBoost feature importance for glaucoma versus control classification. Each point represents one sample; color indicates feature value (blue = low, red = high). Min GCC, Avg GCC, and macular perfusion/vessel density are the dominant discriminators, consistent with the well-established structural–vascular phenotype of glaucoma.

**Figure 5:**
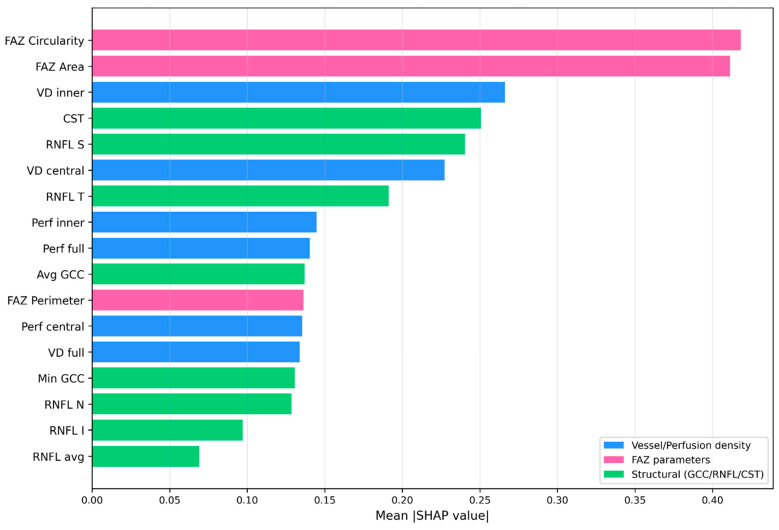
XGBoost feature importance for glaucoma subtype classification (mean |SHAP| across all three classes). FAZ Circularity and FAZ Area are the top two features, supporting FAZ morphology as the dominant macular OCTA biomarker for inter-subtype discrimination. Color coding: blue = vessel/perfusion density; pink = FAZ parameters; green = structural (GCC/RNFL/CST).

SHAP-based feature importance at the cohort level is visualized in Figure 4 (glaucoma vs. control) and Figure 5 (subtype classification). FAZ Circularity (mean |SHAP| = 0.418) and FAZ Area (0.411) were the top two features for subtype classification, followed by VD Inner (0.266) and CST (0.251). These results converge with the Kruskal–Wallis and ROC analyses, supporting FAZ morphology as the dominant macular OCTA feature class for inter-subtype discrimination in this dataset.

To complement the cohort-level SHAP analysis, representative individual cases from each glaucoma subtype were examined to visualize how the classifier assigns a prediction to a specific patient. Figure S1 (Supplementary Materials) presents local SHAP force plot–style bar charts for three cases—one per subtype—selected as the examples with the highest model probability for the correct subtype class. For the POAG case, VD Inner was the dominant positive contributor; for the PXG case, FAZ Circularity, FAZ Area, and Perfusion Inner collectively pushed the prediction toward the correct class, reflecting the combined macular perfusion and geometric FAZ signature observed in pseudoexfoliative disease; and for the NTG case, FAZ Area alone was the dominant positive contributor, consistent with an enlarged foveal avascular zone as a feature of normal-tension glaucoma. These case-level explanations indicate that the global ranking of FAZ morphology is supported by genuine per-patient contributions and is not solely an averaging artifact within this dataset.

## 4. Discussion

This study evaluated macular OCT angiography (OCTA) parameters obtained with a 3 × 3 mm scan protocol in three open-angle glaucoma subtypes—POAG, PXG, and NTG—and compared them with structural OCT and healthy controls. Two interpretive caveats apply throughout. First, the design is single-center, cross-sectional, and case–control, allowing associative but not causal inference; subtype-related patterns described as “vascular signatures” or as “characteristic” of a subtype are cross-sectional phenotypic descriptions rather than evidence of pathophysiological mechanism in the individual patient. Second, the NTG subgroup (*n* = 28), although larger than in many published OCTA studies of normal-tension glaucoma, is the smallest cohort; subtype-stratified inferences involving NTG should be regarded as exploratory and hypothesis-generating. With these caveats, the primary hypothesis—that macular OCTA provides subtype-associated microvascular information complementary to structural imaging—was supported across all analytical dimensions: Kruskal–Wallis comparisons, pairwise ROC discrimination, multivariate regression, and exploratory machine-learning feature importance using SHAP values. Four major findings warrant focused discussion.

The most consistent between-subtype finding was significantly reduced macular VD Inner (16.37 ± 3.33%) and Perfusion Inner (0.31 ± 0.05) in PXG, lower than in POAG (both *p* < 0.001) and NTG (Perfusion Inner *p* < 0.05). The PXG < NTG < POAG gradient held across all three vessel/perfusion parameters (central, inner, full), consistent with a robust, scan-independent phenomenon rather than a regional artifact, and aligns with prior evidence that PXG involves disproportionately severe macular microvascular compromise relative to neuroretinal damage.

Subasi et al., comparing 36 POAG and 34 PXG eyes matched for mean deviation, found that all macular superficial vessel density parameters were lower in PXG, with a statistically significant decrease in the parafoveal region (*p* = 0.008), supporting additional IOP-independent vascular pathology in pseudoexfoliative disease [6]. Cornelius et al. similarly reported significantly lower mean superficial perifoveal perfusion density in PXG than in POAG, indicating perfusion deficits exceeding what IOP-related axonal damage alone would predict [19]. Two further severity-matched analyses converge on the same conclusion and are particularly relevant given that our subtypes were not matched a priori for MD: Naderi Beni et al. found significantly lower macular vessel density in PXG than in POAG at matched functional severity, with a weaker macular VD–functional correlation in PXG, supporting the IOP-independent vascular component [21]; Aghsaei Fard and Ritch likewise observed greater variability in the VD–function relationship in PXG, reflecting partial independence of mechanical and vascular pathways [22]. The pattern in our cohort—pronounced macular vessel and perfusion compromise in PXG, qualitatively distinct from NTG (which has comparable mean MD), and paralleled by weaker macular VD–GCC coupling in PXG than in NTG—is consistent with these severity-matched reports and supports an interpretation beyond a simple severity-confounding explanation.

The pathophysiological basis is well described. Pseudoexfoliative material accumulates in the walls of retinal and choroidal blood vessels, producing endothelial dysfunction, reduced nitric oxide bioavailability, increased plasma endothelin-1, and impaired autoregulation—mechanisms that operate independently of IOP elevation. Chatziralli et al., in a review of 17 studies, concluded that PXG eyes consistently show decreased peripapillary and macular vessel density compared with controls, and that the discrepancy between PXG and POAG in macular VD likely reflects an additional pathological component of the vascular endothelium unique to pseudoexfoliation syndrome [5]. Bourouki et al. demonstrated measurable vascular endothelial dysfunction and arterial stiffness in PXG patients, with elevated circulating apoptotic endothelial microparticles, providing direct mechanistic evidence for the systemic vascular component [9]. The cross-sectional pattern in our cohort is compatible with this published mechanistic framework, although our design cannot test the mechanism itself.

An important nuance from our ROC analysis is that the OCTA-only model for POAG vs. PXG (AUC = 0.695) significantly outperformed the structural-only model (AUC = 0.531; DeLong *p* = 0.002). The extended ROC analysis shows that the three top-performing individual parameters—Perfusion Inner (AUC = 0.706), VD Inner (AUC = 0.705), and FAZ Circularity (AUC = 0.703)—are virtually indistinguishable. This near-equivalence is clinically meaningful: PXG is best characterized not by a single dominant biomarker but by a composite phenotype combining macular perfusion reduction with FAZ geometric distortion. At the same time, absolute AUC differences of 0.10–0.16 between OCTA-based and structural-only models, although statistically significant, correspond to modest improvements at the individual-eye level; clinical translation will depend on prospective validation. This parallel contribution of perfusion and morphology aligns with Safizadeh et al., who, in advanced POAG and PXG, found no significant differences in structural parameters (RNFL, GCC) between groups, yet PXG eyes still showed lower parafoveal vessel density—consistent with vascular parameters discriminating between subtypes precisely when structural parameters cannot [7]. The clinical implication, pending prospective confirmation, is that in a patient presenting with open-angle glaucoma at comparable structural severity, macular OCTA provides multiple, mutually reinforcing vascular signals that may help distinguish IOP-dependent and IOP-independent pathogenic contributions.

By contrast, Rebolleda et al. reported no significant differences in peripapillary or superficial macular vessel density between age- and severity-matched PXG and POAG groups using AngioVue (RTVue-XR), suggesting that device-specific algorithms may influence whether vascular differences are detected [8]. Use of the Cirrus AngioPlex 3 × 3 mm protocol may have increased sensitivity to inner-macular differences in our cohort.

The second major finding was an enlarged FAZ Area (0.27 ± 0.11 mm^2^) in NTG relative to PXG (0.19 ± 0.08 mm^2^, *p* < 0.01) and POAG, along with significantly lower FAZ Circularity in PXG (0.54 ± 0.11) than in POAG (0.60 ± 0.12; *p* < 0.001). FAZ enlargement in NTG occurred despite structurally comparable RNFL and GCC values between POAG and NTG—a pattern consistent with a foveal microvascular signature that is partially independent of neuroretinal thinning rather than a consequence of more advanced structural damage.

The foveal avascular zone, which depends on the surrounding superficial capillary plexus for metabolic support, is sensitive to ischemic insults. Zivkovic et al. were among the first to systematically quantify FAZ dimensions in NTG using OCTA on the Zeiss AngioPlex with a 3 × 3 mm scan—the same instrument and protocol used here—and demonstrated significantly enlarged FAZ vertical, horizontal, and maximum diameters, as well as FAZ area, in NTG patients compared with healthy controls (all *p* < 0.001), attributing this to primary foveal capillary dropout from vascular dysregulation [12]. Our findings reproduce this observation and extend it to a three-way subtype comparison.

Cheng et al., analyzing macular vessel branching complexity in NTG, found that mean deviation decreased by 0.4 dB for each 1% reduction in perifoveal vessel density (95% CI 0.1–0.6 dB, *p* = 0.007) [13]. They also observed a lower multispectral fractal dimension in eyes with systemic hypertension—suggesting that FAZ-related capillary dropout in NTG is modulated by systemic vascular risk factors beyond IOP. This is consistent with the primary vascular dysregulation hypothesis of NTG (Flammer syndrome), in which nocturnal hypotension, vasospasm, and impaired autoregulation drive foveal ischemia–reperfusion injury independently of daytime IOP.

The diagnostic relevance of FAZ enlargement is supported by independent longitudinal and post-surgical evidence. Nishida et al. demonstrated in 115 eyes from glaucoma suspects and open-angle glaucoma patients that FAZ area enlargement over follow-up was associated with glaucoma progression [23]; our cross-sectional design cannot claim temporal precedence. Shoji et al., in a prospective study of 54 POAG patients undergoing trabeculectomy, demonstrated that FAZ area decreased significantly after IOP-lowering surgery (from 0.485 ± 0.193 to 0.446 ± 0.174 mm^2^, *p* < 0.001) and correlated significantly with preoperative foveal sensitivity and the magnitude of IOP reduction after age adjustment [14]. Although NTG is defined by daytime IOP ≤ 21 mmHg, nocturnal IOP fluctuations and reductions in ocular perfusion pressure may contribute to cumulative foveal ischemia, providing a plausible context for FAZ enlargement in this subtype.

Of particular clinical relevance is the finding that FAZ circularity was significantly lower in PXG (0.54 ± 0.11) than in POAG (0.60 ± 0.12; *p* < 0.001). Reduced FAZ circularity reflects irregular perfusion loss within the foveal capillary network beyond what FAZ area enlargement captures. The combination of enlarged FAZ area in NTG and reduced FAZ circularity in PXG is consistent with two distinct patterns: more diffuse capillary dropout producing relatively uniform zone expansion in NTG, versus more irregular perfusion loss producing FAZ shape distortion in PXG.

A key analytical contribution is the subtype-stratified Spearman correlation analysis, which revealed that the relationship between macular vessel density and structural parameters (GCC, RNFL) varies across subtypes. In NTG, VD Inner showed the strongest correlations (vs. Avg GCC: r = 0.52, *p* = 0.005; vs. RNFL avg: r = 0.59, *p* < 0.001), although, given *n* = 28, these point estimates are imprecise. Correlations were intermediate in PXG (r = 0.35 and r = 0.36; both *p* < 0.01) and weakest in POAG (r = 0.40 with Avg GCC, *p* < 0.001; r = 0.23 with RNFL avg, *p* < 0.05).

Jeon et al. demonstrated in 116 POAG eyes that macular vessel density, GCC, and RNFL are significantly correlated (VD vs. GCC: r = 0.48, *p* < 0.0001), with a weaker correlation between macular vessel density and MD (r = 0.3, *p* = 0.0028) than between GCC and MD (r = 0.6, *p* < 0.0001) [17], establishing partial structural–vascular coupling. Our subtype-stratified analysis is consistent with this picture and adds the observation that coupling strength varies by subtype—tightest in NTG and weaker in POAG and PXG. Belghith et al., evaluating circumpapillary vessel density, RNFL, and GCC in healthy, preperimetric, and manifest glaucoma eyes, found that vascular changes appeared secondary to RNFL and GCC damage in POAG (best regression R^2^ = 0.752 for RNFL) [24]. Our finding of a stronger VD–structure correlation in NTG than in POAG is compatible with co-evolution driven by a shared upstream vascular contribution in NTG, whereas the intermediate correlations in PXG are compatible with partial independence of vascular and structural deterioration—although a cross-sectional design cannot distinguish between these explanations and alternatives such as differential measurement noise or selection across the severity spectrum.

Beyond overall coupling strength, the sector-resolved correlation matrix (Supplementary Table S2) reveals topographic heterogeneity that is clinically informative and not apparent from global RNFL measurements alone. In NTG, macular VD Inner and Perfusion Inner correlated most strongly with the RNFL inferior sector (r = 0.53 and r = 0.52, respectively; both *p* < 0.01), whereas correlations with the superior, nasal, and temporal sectors did not reach significance. This inferior-predominant coupling aligns with the well-characterized predilection of NTG for inferotemporal RNFL defects and paracentral visual field loss. In PXG, the strongest sectoral coupling shifted to the RNFL nasal sector (VD Inner vs. RNFL N: r = 0.41, *p* < 0.001; Perfusion Inner vs. RNFL N: r = 0.46, *p* < 0.001), mirroring the sectoral distribution of vascular density reduction reported by Eslami et al. in advanced PXG, in which eyes showed selectively lower vessel density in nasal and inferior parafoveal quadrants compared with matched POAG controls [25]. Faria Pereira et al. similarly identified nasal macular sectors as the preferential location for early glaucomatous change in pseudoexfoliation syndrome, with nasal-inferior and nasal-superior ganglion cell layer thickness reduced in pseudoexfoliative eyes compared with controls, even before glaucoma conversion [26]. Together, these converging cross-sectional observations suggest that the macular vascular profile of PXG may be both quantitatively distinct (lower VD and Perfusion Inner) and topographically distinct (nasal-predominant coupling), warranting prospective investigation as a potential biomarker for subtype-specific monitoring.

FAZ parameters showed no significant correlation with structural parameters across any subtype (all *p* > 0.10), supporting their structural independence. In 95 eyes with early glaucoma followed for a mean of 3.8 years, Mohammadzadeh et al. found that rates of macular VD loss and GCC thinning were both associated with central visual field loss but followed different trajectories, with VD loss often preceding measurable GCC thinning in the earliest stages [18]. The structural independence of FAZ metrics observed here therefore identifies a dimension of foveal vascular pathology that GCC and RNFL measurements do not appear to capture.

Combined OCTA–structural models outperformed structural-only models across all three pairwise comparisons (DeLong *p* < 0.05 in two: POAG vs. PXG and PXG vs. NTG; the POAG vs. NTG comparison did not reach the conventional threshold). For PXG vs. NTG, both OCTA-only (AUC = 0.765) and combined (AUC = 0.770) models outperformed the structural reference (AUC = 0.617), with the combined model achieving DeLong *p* = 0.010. For POAG vs. NTG, the structural-only model performed near chance (AUC = 0.567), whereas the combined model reached AUC = 0.667. This pattern is consistent with the overlapping macular structural and vascular phenotypes of these two subtypes, with the discriminatory signal residing primarily in FAZ morphology rather than in vessel density.

Łukasik et al., comparing early NTG and high-tension PXG using both OCT and OCTA, reported that structural OCT alone fails to distinguish these subtypes in early stages and that OCTA provides complementary discriminatory information when IOP-independent mechanisms are operative [4]. This aligns with our POAG vs. NTG comparison, where FAZ parameters—rather than vessel density—provide the dominant discriminatory signal: in NTG, foveal capillary compromise can be detectable without the peripapillary VD reduction that characterizes more advanced disease.

The additive value of combining OCTA with structural OCT is further supported by Hsia et al., who, in advanced and severe open-angle glaucoma, found that reduced macular superficial and deep vessel densities were independently associated with decreased visual acuity, regardless of age and axial length [27]. Our combined model formalizes an analogous incremental contribution in the inter-subtype discrimination setting using cross-validated logistic regression and DeLong testing.

Across the three subtypes, structural parameters (RNFL avg and Avg GCC) were the most consistent independent predictors of MD in multivariate regression. RNFL avg reached significance in all three subtypes (POAG β = 0.128, *p* = 0.002; PXG β = 0.154, *p* = 0.004; NTG β = 0.190, *p* = 0.031), and Avg GCC was significant in PXG (β = 0.189, *p* = 0.004), with borderline trends in POAG and NTG.

A novel finding was that Perfusion Inner emerged as an independent predictor of MD in PXG (β = −32.783, 95% CI −62.75 to −2.82, *p* = 0.032), whereas macular OCTA parameters did not reach significance in POAG or NTG. This subtype-specific significance aligns with prior reports describing pseudoexfoliative disease as involving a vascular endothelial component that contributes to ganglion-cell damage alongside mechanical trabecular obstruction [5,9]. Our finding that macular perfusion captures additional predictive information for visual field loss specifically in PXG provides cross-sectional support for this published framework and suggests that macular OCTA perfusion may have particular value for prospective evaluation of disease activity and progression risk in pseudoexfoliative glaucoma.

The non-significance of OCTA parameters in MD regression for POAG and NTG should not be interpreted as a failure of macular OCTA. RNFL and GCC measure neuroretinal tissue, and their thinning translates directly into loss of visual field sensitivity, making the structure–function relationship mechanistically direct. In 84 POAG eyes, Dhabarde et al. confirmed that the GCC–MD relationship is best described by a curvilinear (second-order polynomial) function (*p* < 0.001), with GCC showing diagnostic value comparable to RNFL across all stages [28]. Macular vessel density, by contrast, reflects the capillary network perfusing ganglion cells—a parameter whose relationship with visual function is mediated by tissue thinning rather than a direct sensory pathway.

The longitudinal evidence cited above [18] frames the lack of OCTA significance in POAG and NTG regressions as consistent with a complementary rather than redundant role: capturing vascular pathology that may operate upstream of the neuroretinal thinning driving current perimetric loss. The emergence of Perfusion Inner as a significant predictor, specifically in PXG, is consistent with this upstream vascular signal being more closely coupled to the functional pathway in pseudoexfoliative disease.

The exploratory XGBoost classifier achieved an internal 5-fold cross-validated AUC of 0.975 ± 0.008 for glaucoma vs. control discrimination, consistent with the strong collective discriminatory signal of the combined OCTA and structural feature set. This figure reflects internal 5-fold cross-validation on a single dataset and is expected to overstate performance in independent populations; we explicitly do not present it as an estimate of generalizable diagnostic accuracy. For glaucoma subtype classification, 5-fold cross-validated accuracy was 0.667 ± 0.054. SHAP-based feature importance identified FAZ Circularity (mean |SHAP| = 0.418) and FAZ Area (mean |SHAP| = 0.411) as the top two features for subtype classification, ranking virtually identically and substantially ahead of VD Inner (0.266) and CST (0.251)—supporting FAZ morphology as the dominant inter-subtype feature class.

Advances in OCT and OCTA imaging have broadened the set of features available for machine-learning-based classification, as summarized by Ong et al. [29]. Feature importance is task-dependent: for glaucoma vs. healthy discrimination, peripapillary and macular vessel density typically dominate; for inter-subtype discrimination—our task—FAZ morphology dominates because it captures the foveal vascular dimension, which is abnormal in NTG and geometrically distorted in PXG. Kim et al. reported XGBoost accuracies of 0.903–0.947 in an explainable model combining visual field, RNFL OCT, and IOP, and confirmed that SHAP visualizations provided clinically interpretable explanations [30]. Our application of the same interpretability framework to OCTA-based subtype classification extends this to the more challenging problem of inter-subtype discrimination, while explicitly acknowledging that no held-out external test set was available.

Cohort-level rankings can mask heterogeneity in how a classifier reaches a decision for each case. To address this, we examined local SHAP contributions for one representative patient per subtype, selected as the case with the highest model probability for the correct class. For the POAG case, VD Inner was the dominant positive contributor; for the PXG case, FAZ Circularity, FAZ Area, and Perfusion Inner collectively pushed the prediction toward the correct class, reflecting the combined macular perfusion and geometric FAZ signature of pseudoexfoliative disease; for the NTG case, FAZ Area alone was the dominant contributor. These case-level explanations indicate that the global ranking of FAZ morphology is supported by genuine per-case contributions rather than an averaging artifact. The capability for case-level interpretability aligns with the workflow of Kim et al., in which SHAP local explanations allow patient-by-patient inspection [30]—essential for any future clinical deployment, because cohort-level accuracy does not guarantee defensible individual predictions and, in our case, also does not generalize beyond the cross-validation procedure used. Convergence of Kruskal–Wallis, ROC, regression, and global-plus-local SHAP analyses—all pointing to FAZ morphology as the dominant inter-subtype feature—provides triangulated within-cohort evidence; external validation is required.

Oh et al. developed an artificial neural network model using Spectralis OCT parameters that successfully differentiated open-angle glaucoma from glaucoma-suspect eyes without requiring a visual field test [31], demonstrating the diagnostic potential of machine-learning approaches applied to OCT-derived features alone. Our XGBoost-with-SHAP approach builds on this trajectory by combining structural and vascular OCTA features and providing per-feature attributions for each prediction.

A distinguishing methodological feature is the exclusive use of a 3 × 3 mm macular OCTA scan centered on the fovea, which captures the superficial capillary plexus at the highest spatial resolution achievable on the AngioPlex platform, enabling precise FAZ delineation and quantification of inner macular vessel density. Its limitation relative to the 6 × 6 mm scan is the exclusion of the perifoveal zone; its advantage is superior central foveal resolution, where FAZ changes are most diagnostically informative for NTG, and it provides direct methodological comparability to the original validation by Zivkovic et al. [12].

Several limitations must be acknowledged. First, the design is single-center, cross-sectional, and case–control. This precludes longitudinal assessment of whether the subtype-associated OCTA patterns identified here predict differential rates of structural and functional progression and limit causal inference. Cross-sectional associations of “vascular signatures” with subtypes should not be interpreted as evidence of a specific pathophysiological mechanism. Recruitment from a single specialist hospital also constrains generalizability. Prospective, multicenter, longitudinal studies with serial 3 × 3 mm macular OCTA imaging are needed.

Second, although the NTG subgroup (*n* = 28) is larger than in many published OCTA studies of normal-tension glaucoma, it remains underpowered for some subgroup analyses; inferences specific to NTG, including subgroup regressions and pairwise comparisons involving NTG, are reported as exploratory. The strong VD–structure correlations observed in NTG should be interpreted with caution, given the imprecision of these correlations at this sample size.

Third, several potential confounders of OCTA-derived vascular parameters were not formally modeled. IOP itself is intrinsically tied to subtype assignment (NTG defined by IOP ≤ 21 mmHg, POAG and PXG by elevated IOP); statistical adjustment for IOP would absorb the very dimension of variation that defines the subtypes and would not constitute meaningful covariate adjustment. Although patients with documented systemic vascular comorbidities were excluded, structured numerical data on systemic hypertension severity, antihypertensive therapy, and other vascular comorbidities were not extracted for analytical adjustment. Detailed drug-class data on topical antiglaucoma therapy—which differs in its known effects on ocular blood flow among prostaglandin analogues, beta-blockers, alpha-2 agonists, and carbonic anhydrase inhibitors—were not retrievable in structured form across the entire dataset; given a single institutional protocol with prostaglandin analogues as the dominant first-line agent, systematic drug-class confounding would be expected to operate similarly across subtypes, but a residual differential effect cannot be excluded. Additionally, the three subtypes were not matched a priori for severity; PXG had the most negative MD (−6.6 ± 6.9 dB), so some component of lower vessel and perfusion density in PXG could reflect more advanced disease. However, NTG had a comparable mean MD (−6.3 ± 5.2 dB) yet differed markedly from PXG in OCTA profile, and the FAZ findings in NTG occur at structural parameters that are at most modestly worse than in POAG, limiting the plausibility of severity confounding as the sole driver of the subtype-specific patterns. Healthy controls were screened for normal IOP, normal optic nerve and visual field, no family history of glaucoma, and absence of systemic vascular disease, but additional structured vascular-risk assessment was not performed.

Fourth, the statistical strategy comprised multiple analytical streams. Within each stream, conventional control of type I error was applied (Bonferroni correction for the three pairwise post hoc comparisons after Kruskal–Wallis; DeLong tests for ROC model comparisons; standard regression inference). However, we did not apply a global family-wise error rate correction across all aims, and findings based on a single aim or marginal *p*-values—particularly within NTG—should be regarded as exploratory and hypothesis-generating.

Fifth, because only a 3 × 3 mm macular scan was used, peripapillary OCTA parameters were not measured, precluding direct comparison with studies using optic nerve head or peripapillary protocols.

Sixth, the XGBoost classification results are exploratory: 5-fold cross-validation on a single dataset, with no held-out test set and no external validation. The reported AUC of 0.975 ± 0.008 should be treated as an internal estimate likely to be optimistic relative to performance in independent populations, and the SHAP feature rankings as data-driven hypotheses for prospective evaluation, not as evidence supporting clinical deployment.

Seventh, although the great majority of patients in this cohort contributed only one eye to the analytical dataset—with only six patients (≈3% of the glaucoma cohort) contributing both eyes—primary analyses were conducted at the eye level rather than using formal mixed-effects or generalized estimating equation models to account for inter-eye correlation. The pre-specified one-eye-per-patient sensitivity analysis (Section 2.5; Results Sensitivity Analysis: One Eye per Patient) confirmed concordance with the principal findings, including all between-subtype Kruskal–Wallis comparisons, the pairwise ROC discrimination patterns, and the regression-derived prediction of MD by Perfusion Inner in PXG. Residual bias from intra-cluster correlation in full-cohort analyses is expected to be minimal; prospective replication studies should adopt a one-eye-per-patient design at enrollment or apply mixed-effects modeling.

Despite these limitations, the clinical implications of our cross-sectional findings appear coherent and warrant prospective investigation: macular OCTA and FAZ morphology in particular, provide information not accessible through standard structural imaging and are candidate components of comprehensive glaucoma subtype assessment in future longitudinal and externally validated studies.

## 5. Conclusions

In this single-center, cross-sectional cohort of 304 eyes, macular OCTA (3 × 3 mm protocol) revealed subtype-associated microvascular profiles in open-angle glaucoma that were complementary to, not redundant with, structural OCT. PXG showed the most pronounced reductions in macular vessel and perfusion densities, and macular Perfusion Inner additionally predicted MD in this subtype. NTG was distinguished by an enlarged, less circular FAZ in the absence of a proportional reduction in macular vessel density. Combined OCTA–structural multivariable models outperformed structural-only models for the POAG vs. PXG and PXG vs. NTG comparisons, while sector-resolved correlation analysis suggested topographically distinct vascular–structural coupling—inferior-predominant in NTG and nasal-predominant in PXG. An exploratory XGBoost/SHAP analysis identified FAZ Circularity and FAZ Area as the dominant inter-subtype features, with case-level explanations supporting the global ranking. These findings support prospective evaluation of macular OCTA, and of FAZ morphology in particular, as a candidate adjunct to structural OCT in subtype-guided glaucoma assessment. External validation in independent, longitudinal cohorts is required before clinical deployment.

## Figures and Tables

**Table 1:** Demographic and clinical characteristics by glaucoma subtype.

Characteristic	Control (*n* = 106)	POAG (*n* = 102)	PXG (*n* = 68)	NTG (*n* = 28)
Age (years), mean ± SD	64.4 ± 10.6	64.6 ± 6.8	66.7 ± 7.2	65.3 ± 7.0
Sex (female), *n* (%)	62 (58.5%)	56 (54.9%)	41 (60.3%)	17 (60.7%)
MD (dB), mean ± SD	0.44 ± 1.28	−5.0 ± 5.1	−6.6 ± 6.9	−6.3 ± 5.2
PSD (dB), mean ± SD	1.68 ± 0.38	4.3 ± 2.8	4.1 ± 1.8	7.3 ± 1.5

Values are mean ± SD unless otherwise stated. POAG = primary open-angle glaucoma (*glaucoma simplex*); PXG = pseudoexfoliative glaucoma (*glaucoma capsulare*); NTG = normal-tension glaucoma (*glaucoma sine tensio*); MD = mean deviation; PSD = pattern standard deviation.

**Table 2:** Macular OCTA and structural parameters by glaucoma subtype: mean ± SD and pairwise statistical comparisons.

Parameter	Control (*n* = 106)	POAG (*n* = 102)	PXG (*n* = 68)	NTG (*n* = 28)	KW *p*	Post Hoc †
***OCT Angiography—Vessel Density***						
VD Central (%)	11.41 ± 2.82	9.22 ± 3.30	7.51 ± 3.48	8.29 ± 3.80	0.004	POAG > PXG **
VD Inner (%)	22.01 ± 1.54	18.73 ± 3.41	16.37 ± 3.33	17.99 ± 2.79	<0.001	POAG > PXG ***
VD Full (%)	20.85 ± 1.59	17.66 ± 3.24	15.52 ± 3.30	16.94 ± 2.76	<0.001	POAG > PXG ***
***OCT Angiography—Perfusion Density***						
Perf Central	0.20 ± 0.06	0.16 ± 0.06	0.14 ± 0.06	0.15 ± 0.07	0.028	POAG > PXG *
Perf Inner	0.40 ± 0.03	0.34 ± 0.05	0.31 ± 0.05	0.34 ± 0.05	<0.001	POAG > PXG ***; PXG < NTG *
Perf Full	0.38 ± 0.03	0.32 ± 0.05	0.29 ± 0.05	0.31 ± 0.04	<0.001	POAG > PXG ***
***FAZ Morphology***						
FAZ Area (mm^2^)	0.27 ± 0.08	0.23 ± 0.11	0.19 ± 0.08	0.27 ± 0.11	<0.001	POAG > PXG **; PXG < NTG **
FAZ Perimeter (mm)	2.18 ± 0.35	2.12 ± 0.59	1.99 ± 0.53	2.39 ± 0.51	0.007	PXG < NTG *
FAZ Circularity	0.67 ± 0.10	0.60 ± 0.12	0.54 ± 0.11	0.57 ± 0.08	<0.001	POAG > PXG ***
***Structural Parameters—GCC***						
Avg GCC (μm)	86.01 ± 3.46	72.90 ± 12.39	71.13 ± 12.76	77.68 ± 9.85	0.043	PXG < NTG *
Min GCC (μm)	82.55 ± 1.70	66.22 ± 15.69	65.06 ± 14.47	72.04 ± 11.15	0.083	ns
***Structural Parameters—RNFL***						
RNFL avg (μm)	97.76 ± 10.12	81.52 ± 13.13	77.18 ± 15.56	85.07 ± 11.74	0.029	ns
RNFL Superior (μm)	120.37 ± 17.05	98.73 ± 19.68	89.06 ± 25.92	104.00 ± 17.92	0.012	POAG > PXG *; PXG < NTG *
RNFL Nasal (μm)	75.02 ± 11.52	67.27 ± 10.96	67.66 ± 17.73	68.93 ± 10.83	0.407	ns
RNFL Inferior (μm)	127.55 ± 14.82	100.08 ± 25.15	95.28 ± 25.34	105.18 ± 27.64	0.233	ns
RNFL Temporal (μm)	67.36 ± 10.53	60.26 ± 11.46	59.06 ± 11.72	61.71 ± 6.32	0.335	ns
***Other***						
CST (μm)	257.94 ± 18.31	260.59 ± 24.46	260.51 ± 32.35	261.07 ± 26.78	0.866	ns
MD (dB)	0.44 ± 1.28	−4.97 ± 5.14	−6.58 ± 6.94	−6.31 ± 5.24	0.026	ns
PSD (dB)	1.68 ± 0.38	4.28 ± 2.76	4.08 ± 1.76	7.27 ± 1.54	<0.001	POAG < NTG ***; PXG < NTG ***

KW = Kruskal–Wallis; * *p* < 0.05; ** *p* < 0.01; *** *p* < 0.001. For MD and PSD, control values are shown for descriptive reference; statistical comparisons in the post hoc column refer to the three glaucoma subtypes only, † Post hoc pairwise comparisons performed using Mann–Whitney U tests with Bonferroni correction for three comparisons.

**Table 3:** Spearman correlations between macular OCTA parameters and structural parameters by glaucoma subtype.

OCTA Parameter	Structural	All r/*p*	POAG r/*p*	PXG r/*p*	NTG r/*p*	Interpretation
***Vessel Density Inner***						
VD Inner	Avg GCC	0.41/<0.001 †	0.40/<0.001 †	0.35/0.003 †	0.52/0.005 †	Significant in all subtypes
VD Inner	RNFL avg	0.34/<0.001 †	0.23/0.022 †	0.36/0.003 †	0.59/<0.001 †	Strongest in NTG
***Perfusion Inner***						
Perf Inner	Avg GCC	0.40/<0.001 †	0.40/<0.001 †	0.30/0.013 †	0.48/0.009 †	Same pattern as VD Inner
Perf Inner	RNFL avg	0.32/<0.001 †	0.22/0.025 †	0.33/0.007 †	0.56/0.002 †	Moderate overall
***FAZ Parameters***						
FAZ Area	Avg GCC	−0.01/0.944	−0.04/0.719	0.03/0.788	−0.31/0.109	No significant correlation
FAZ Area	RNFL avg	−0.13/0.063	−0.22/0.023 †	−0.09/0.489	−0.36/0.061	Weak inverse in POAG only
FAZ Perimeter	Avg GCC	−0.06/0.407	−0.04/0.695	−0.08/0.493	−0.30/0.119	ns across all subtypes
FAZ Perimeter	RNFL avg	−0.19/0.008 †	−0.26/0.008 †	−0.17/0.175	−0.36/0.062	POAG only

r = Spearman correlation coefficient; *p* = two-sided *p*-value. † *p* < 0.05.

**Table 4:** ROC analysis: individual parameters and combination models for pairwise discrimination between glaucoma subtypes.

Parameter/Model	*n*	AUC	95% CI	Sens.	Spec.	*p* (DeLong vs. Struct.)	Type
***POAG vs. PXG***							
Perf Inner (best individual)	170	0.706	0.626–0.785	75%	63%	–	OCTA
VD Inner	170	0.705	0.626–0.784	64%	72%	–	OCTA
FAZ Circularity	170	0.703	0.621–0.784	66%	82%	–	OCTA
RNFL avg	170	0.597	0.509–0.685	73%	47%	–	Structural
Avg GCC	170	0.548	0.458–0.638	67%	46%	–	Structural
Structural (GCC + RNFL)	170	0.531	0.441–0.620	63%	51%	ref.	Combined
**OCTA only**	170	0.695	0.615–0.774	70%	60%	0.002	Combined
**Combined (Angio + Structural)**	170	0.684	0.603–0.765	67%	63%	0.002	Combined
***POAG vs. NTG***							
FAZ Perimeter (best individual)	130	0.619	0.496–0.742	75%	57%	–	FAZ
FAZ Area	130	0.612	0.490–0.734	72%	61%	–	FAZ
Avg GCC	130	0.619	0.499–0.740	82%	43%	–	Structural
RNFL avg	130	0.555	0.439–0.671	27%	89%	–	Structural
Structural (GCC + RNFL)	130	0.567	0.446–0.689	57%	61%	ref.	Combined
OCTA only	130	0.563	0.436–0.690	94%	21%	0.967	Combined
Combined (Angio + Structural)	130	0.667	0.552–0.781	64%	79%	0.171	Combined
***PXG vs. NTG***							
FAZ Area (best individual)	96	0.701	0.568–0.834	87%	61%	–	FAZ
FAZ Perimeter	96	0.670	0.540–0.799	79%	61%	–	FAZ
Avg GCC	96	0.659	0.540–0.778	69%	57%	–	Structural
RNFL avg	96	0.651	0.537–0.765	47%	89%	–	Structural
Structural (GCC + RNFL)	96	0.617	0.495–0.738	53%	75%	ref.	Combined
OCTA only	96	0.765	0.661–0.869	74%	79%	0.060	Combined
**Combined (Angio + Structural)**	96	0.770	0.667–0.873	72%	75%	0.010	Combined

AUC = area under the ROC curve; 95% CI by DeLong method. Sensitivity and specificity are reported at the Youden index–optimal threshold. Combination models: 5-fold cross-validated logistic regression (L2, C = 0.1). DeLong test compares each model versus the structural-only reference. Bold rows indicate combination models that significantly outperform the structural-only reference (*p <* 0.05).

**Table 5:** Multivariate OLS regression: OCT parameters as predictors of MD (dB), adjusted for age and sex.

Predictor	*n*	β	95% CI	*p*	Adj. R^2^ (Δ)
***POAG (n = 102)***					
**RNFL avg**	102	0.128	0.049–0.208	0.002 **	0.085
Avg GCC	102	0.082	−0.005–0.170	0.064	0.025
VD Inner	102	0.222	−0.089–0.533	0.160	0.010
Perf Inner	102	13.704	−5.886–33.294	0.168	0.009
FAZ Area	102	5.577	−4.069–15.224	0.254	0.003
***PXG (n = 68)***					
**RNFL avg**	68	0.154	0.052–0.255	0.004 **	0.106
**Avg GCC**	68	0.189	0.064–0.313	0.004 **	0.106
VD Inner	68	−0.375	−0.872–0.122	0.136	0.018
**Perf Inner**	68	−32.783	−62.746 to −2.821	0.032 *	0.052
FAZ Area	68	−0.903	−22.383–20.578	0.933	−0.015
***NTG (n = 28) ‡—exploratory***					
**RNFL avg**	28	0.190	0.019–0.362	0.031 *	0.128
Avg GCC	28	0.182	−0.012–0.376	0.065	0.087
VD Inner	28	0.518	−0.167–1.204	0.132	0.048
Perf Inner	28	35.732	−5.078–76.542	0.083	0.073
FAZ Area	28	−1.424	−21.519–18.670	0.885	−0.036

β = unstandardised regression coefficient; Adj. R^2^ (Δ) = additional variance explained by the predictor beyond age and sex. All models include age and sex as covariates. * *p* < 0.05; ** *p* < 0.01. Bold rows indicate statistically significant predictors. ‡ NTG: *n* = 28—all results in this subgroup are exploratory and should be interpreted with caution, given limited statistical power.

## Data Availability

The authors confirm that the data supporting the findings of this study are available within the article.

## Supplementary Materials

The following supporting information can be downloaded at: https://www.mdpi.com/article/10.3390/medicina62050941/s1, Figure S1: SHAP (SHapley Additive exPlanations) feature contribution plots for one representative case from each of the three glaucoma subtypes, selected as the example with the highest model probability for the correct subtype class.; Figure S2: Receiver operating characteristic (ROC) curves for the top five individual parameters (OCTA and structural) in each of the three pairwise glaucoma subtype comparisons.; Table S1: Extended ROC analysis: individual parameter performance for pairwise glaucoma subtype discrimination; Table S2: Extended Spearman correlation matrix: macular OCTA parameters versus structural parameters, by glaucoma subtype; Table S3:Detailed multivariate OLS regression models: individual OCT parameters as predictors of visual field mean deviation; Table S4: Kruskal–Wallis comparisons of macular OCTA and structural parameters across the three glaucoma subtypes: full cohort (n=197) versus one-eye-per-patient subset (n=191); Bonferroni-corrected Mann–Whitney pairwise post hoc comparisons for the principal macular OCTA parameters: full cohort versus one-eye-per-patient subset; 5-fold cross-validated ROC analysis: structural-only, OCTA-only, and combined logistic-regression models across the three pairwise subtype comparisons. Full cohort versus one-eye-per-patient subset; Multivariate OLS regression of mean deviation (MD) on the principal OCTA and structural predictors within each subtype, adjusted for age and sex. Full cohort versus one-eye-per-patient subset.
