# Supplementary material for: Subtype-Specific Macular Vascular Signatures in Primary Open-Angle, Pseudoexfoliative, and Normal-Tension Glaucoma: OCT Angiography Study
Source: Medicina (Kaunas). 2026 May 12;62(5):941. doi: 10.3390/medicina62050941 (PMC13208426; doi:10.3390/medicina62050941)
Supplement: Supplementary file 1 [file medicina-62-00941-s001.zip › medicina-4301539-supplementary.pdf]

Supplementary Materials

Table S1. Extended ROC analysis: individual parameter performance for pairwise glaucoma subtype discrimination.

G. simplex vs G. capsulare (n = 170)

Parameter	AUC	95% CI	Sensitivity	Specificity
Perf Inner	0.706	0.626–0.785	0.75	0.63
VD Inner	0.705	0.626–0.784	0.64	0.72
FAZ Circularity	0.703	0.621–0.784	0.66	0.82
Perf Full	0.690	0.609–0.771	0.78	0.59
VD Full	0.689	0.608–0.770	0.65	0.69
VD Central	0.654	0.569–0.739	0.74	0.54
FAZ Area	0.642	0.556–0.727	0.68	0.72
Perf Central	0.621	0.534–0.708	0.60	0.65
RNFL S	0.613	0.525–0.702	0.75	0.49
FAZ Perimeter	0.600	0.512–0.688	0.64	0.71
RNFL avg	0.597	0.509–0.685	0.73	0.47
RNFL I	0.558	0.470–0.646	0.34	0.81
Avg GCC	0.548	0.458–0.638	0.67	0.46
RNFL N	0.545	0.454–0.635	0.81	0.32
Min GCC	0.540	0.451–0.629	0.70	0.41
RNFL T	0.524	0.433–0.615	0.52	0.59
CST	0.524	0.432–0.615	0.87	0.24

G. simplex vs G. sine tensio (n = 130)

Parameter	AUC	95% CI	Sensitivity	Specificity
FAZ Perimeter	0.619	0.496–0.742	0.75	0.57
Avg GCC	0.619	0.499–0.740	0.82	0.43
FAZ Area	0.612	0.490–0.734	0.72	0.61
FAZ Circularity	0.610	0.503–0.717	0.66	0.64
Min GCC	0.600	0.482–0.718	0.29	0.93
VD Full	0.598	0.483–0.713	0.74	0.46
VD Inner	0.595	0.482–0.708	0.61	0.61
Perf Full	0.579	0.458–0.700	0.77	0.43
Perf Central	0.576	0.442–0.710	0.76	0.46
RNFL T	0.575	0.469–0.681	0.45	0.79
VD Central	0.570	0.438–0.702	0.90	0.32
Perf Inner	0.566	0.447–0.684	0.60	0.57
RNFL avg	0.555	0.439–0.671	0.27	0.89
RNFL S	0.546	0.428–0.664	0.25	0.93
RNFL I	0.542	0.420–0.665	0.55	0.61
RNFL N	0.539	0.416–0.663	0.48	0.64
CST	0.504	0.369–0.639	0.87	0.29

G. capsulare vs G. sine tensio (n = 96)

Parameter	AUC	95% CI	Sensitivity	Specificity
FAZ Area	0.701	0.568–0.834	0.87	0.61
FAZ Perimeter	0.670	0.540–0.799	0.79	0.61
Perf Inner	0.664	0.547–0.780	0.56	0.79
RNFL S	0.663	0.552–0.773	0.50	0.86
Avg GCC	0.659	0.540–0.778	0.69	0.57
RNFL avg	0.651	0.537–0.765	0.47	0.89
Min GCC	0.650	0.532–0.769	0.40	0.93
FAZ Circularity	0.638	0.511–0.766	0.74	0.68
Perf Full	0.638	0.520–0.756	0.43	0.82
VD Inner	0.636	0.518–0.755	0.47	0.79
VD Full	0.623	0.504–0.741	0.38	0.86
RNFL I	0.601	0.475–0.728	0.59	0.61
RNFL T	0.592	0.479–0.705	0.51	0.79
RNFL N	0.578	0.454–0.702	0.47	0.68
VD Central	0.558	0.423–0.693	0.78	0.46
Perf Central	0.523	0.388–0.658	0.91	0.25
CST	0.522	0.391–0.653	0.35	0.79

Area under the receiver operating characteristic curve (AUC) with 95% confidence intervals (DeLong method), sensitivity, and specificity at the optimal (Youden) threshold for all 17 individual OCTA and structural parameters across three pairwise comparisons. Parameters are sorted by AUC within each comparison.

Table S2. Extended Spearman correlation matrix: macular OCTA parameters versus structural parameters, by glaucoma subtype.

G. simplex

OCTA parameter	Avg GCC	Min GCC	RNFL avg	RNFL S	RNFL N	RNFL I	RNFL T
**VD Central**	0.139	0.138	0.286**	0.207*	0.320**	0.193	0.161
**VD Inner**	0.402***	0.384***	0.226*	0.232*	0.136	0.250*	0.013
**VD Full**	0.403***	0.387***	0.263**	0.264**	0.181	0.271**	0.027
**Perf Central**	0.168	0.165	0.285**	0.200*	0.302**	0.211*	0.146
**Perf Inner**	0.405***	0.379***	0.222*	0.230*	0.124	0.237*	0.036
**Perf Full**	0.396***	0.374***	0.251*	0.257**	0.155	0.252*	0.059
**FAZ Area**	-0.036	-0.021	-0.224*	-0.170	-0.332***	-0.128	-0.185
**FAZ Perim**	-0.039	-0.043	-0.261**	-0.199*	-0.314**	-0.201*	-0.160
**FAZ Circ**	0.162	0.211*	0.150	0.162	-0.035	0.207*	-0.037

G. capsulare

OCTA parameter	Avg GCC	Min GCC	RNFL avg	RNFL S	RNFL N	RNFL I	RNFL T
**VD Central**	0.094	0.084	0.111	-0.035	0.338**	0.038	0.286*
**VD Inner**	0.352**	0.388**	0.361**	0.258*	0.408***	0.238	0.269*
**VD Full**	0.367**	0.393***	0.366**	0.250*	0.431***	0.253*	0.308*
**Perf Central**	0.169	0.137	0.240*	0.093	0.352**	0.197	0.252*
**Perf Inner**	0.299*	0.336**	0.325**	0.219	0.456***	0.198	0.265*
**Perf Full**	0.297*	0.322**	0.310*	0.168	0.450***	0.201	0.286*
**FAZ Area**	0.033	0.033	-0.085	-0.147	-0.057	0.072	-0.035
**FAZ Perim**	-0.085	-0.052	-0.167	-0.176	0.032	-0.082	-0.157
**FAZ Circ**	0.103	0.117	0.111	0.107	0.057	0.093	0.054

G. sine tensio

OCTA parameter	Avg GCC	Min GCC	RNFL avg	RNFL S	RNFL N	RNFL I	RNFL T
**VD Central**	0.204	0.193	0.305	0.188	0.173	0.130	-0.057
**VD Inner**	0.517**	0.511**	0.589***	0.259	0.275	0.534**	-0.004
**VD Full**	0.523**	0.507**	0.564**	0.251	0.263	0.504**	-0.023
**Perf Central**	0.209	0.200	0.277	0.135	0.115	0.128	-0.081

**Perf Inner**	0.483**	0.500**	0.559**	0.217	0.219	0.517**	0.087
**Perf Full**	0.513**	0.519**	0.560**	0.225	0.233	0.502**	0.024
**FAZ Area**	-0.309	-0.248	-0.358	-0.475*	-0.308	-0.162	0.215
**FAZ Perim**	-0.302	-0.241	-0.357	-0.399*	-0.326	-0.108	0.222
**FAZ Circ**	0.172	0.135	0.204	-0.214	0.023	0.175	-0.177

*Spearman rank correlation coefficients (r) between 9 macular OCTA parameters and 7 structural parameters (retinal nerve fibre layer and ganglion cell complex metrics) for each glaucoma subtype. Asterisks denote statistical significance: * p<0.05; ** p<0.01; *** p<0.001.*

Table S3. Detailed multivariate OLS regression models: individual OCT parameters as predictors of visual field mean deviation.

G. simplex

Model: MD ~ RNFL avg + age + sex (n = 102)

Variable	β	SE	95% CI	p-value	Significance
Intercept	-18.687	6.787	-32.156, -5.218	0.0070	**
**RNFL avg**	0.128	0.040	0.049, 0.208	0.0017	**
Age (years)	0.040	0.076	-0.112, 0.191	0.6035	n.s.
Sex (female=1)	1.231	0.982	-0.719, 3.180	0.2133	n.s.

Model fit: F = 4.62, p = 0.0046; R^2 = 0.124, adjusted R^2 = 0.097; residual SE = 4.882

Model: MD ~ Avg GCC + age + sex (n = 102)

Variable	β	SE	95% CI	p-value	Significance
Intercept	-11.986	6.848	-25.575, 1.604	0.0832	n.s.
**Avg GCC**	0.082	0.044	-0.005, 0.170	0.0637	n.s.
Age (years)	0.004	0.079	-0.152, 0.160	0.9553	n.s.
Sex (female=1)	1.311	1.024	-0.721, 3.342	0.2034	n.s.

Model fit: F = 2.27, p = 0.0856; R^2 = 0.065, adjusted R^2 = 0.036; residual SE = 5.044

Model: MD ~ VD Inner + age + sex (n = 102)

Variable	β	SE	95% CI	p-value	Significance
Intercept	-9.092	6.546	-22.082, 3.898	0.1680	n.s.
**VD Inner**	0.222	0.157	-0.089, 0.533	0.1596	n.s.
Age (years)	-0.013	0.078	-0.168, 0.142	0.8664	n.s.
Sex (female=1)	1.481	1.022	-0.547, 3.508	0.1506	n.s.

Model fit: F = 1.75, p = 0.1626; R^2 = 0.051, adjusted R^2 = 0.022; residual SE = 5.081

Model: MD ~ Perf Inner + age + sex (n = 102)

Variable	β	SE	95% CI	p-value	Significance
Intercept	-9.373	6.745	-22.759, 4.012	0.1678	n.s.
**Perf Inner**	13.704	9.871	-5.886, 33.294	0.1682	n.s.
Age (years)	-0.017	0.077	-0.171, 0.136	0.8215	n.s.
Sex (female=1)	1.508	1.020	-0.516, 3.532	0.1425	n.s.

Model fit: F = 1.72, p = 0.1682; R^2 = 0.050, adjusted R^2 = 0.021; residual SE = 5.084

Model: MD ~ FAZ Area + age + sex (n = 102)

Variable	β	SE	95% CI	p-value	Significance
Intercept	-3.689	4.956	-13.524, 6.147	0.4585	n.s.

**FAZ Area**	5.577	4.861	-4.069, 15.224	0.2540	n.s.
Age (years)	-0.053	0.075	-0.202, 0.096	0.4831	n.s.
Sex (female=1)	1.547	1.022	-0.482, 3.576	0.1334	n.s.

Model fit: F = 1.51, p = 0.2172; R² = 0.044, adjusted R² = 0.015; residual SE = 5.099

G. capsulare

Model: MD ~ RNFL avg + age + sex (n = 68)

Variable	β	SE	95% CI	p-value	Significance
Intercept	-10.154	8.899	-27.930, 7.623	0.2581	n.s.
**RNFL avg**	0.154	0.051	0.052, 0.255	0.0035	**
Age (years)	-0.098	0.110	-0.318, 0.122	0.3785	n.s.
Sex (female=1)	-2.954	1.585	-6.121, 0.213	0.0670	n.s.

Model fit: F = 5.11, p = 0.0031; R² = 0.193, adjusted R² = 0.156; residual SE = 6.382

Model: MD ~ Avg GCC + age + sex (n = 68)

Variable	β	SE	95% CI	p-value	Significance
Intercept	-12.438	9.352	-31.121, 6.244	0.1882	n.s.
**Avg GCC**	0.189	0.062	0.064, 0.313	0.0036	**
Age (years)	-0.088	0.111	-0.309, 0.133	0.4294	n.s.
Sex (female=1)	-2.790	1.590	-5.967, 0.387	0.0842	n.s.

Model fit: F = 5.10, p = 0.0032; R² = 0.193, adjusted R² = 0.155; residual SE = 6.384

Model: MD ~ VD Inner + age + sex (n = 68)

Variable	β	SE	95% CI	p-value	Significance
Intercept	10.178	8.448	-6.699, 27.055	0.2327	n.s.
**VD Inner**	-0.375	0.249	-0.872, 0.122	0.1363	n.s.
Age (years)	-0.127	0.115	-0.357, 0.103	0.2732	n.s.
Sex (female=1)	-3.525	1.672	-6.866, -0.185	0.0389	*

Model fit: F = 2.61, p = 0.0588; R² = 0.109, adjusted R² = 0.067; residual SE = 6.707

Model: MD ~ Perf Inner + age + sex (n = 68)

Variable	β	SE	95% CI	p-value	Significance
Intercept	12.523	8.325	-4.108, 29.154	0.1374	n.s.
**Perf Inner**	-32.783	14.998	-62.746, -2.821	0.0325	*
Age (years)	-0.103	0.114	-0.331, 0.125	0.3717	n.s.
Sex (female=1)	-3.737	1.647	-7.027, -0.448	0.0266	*

Model fit: F = 3.52, p = 0.0200; R² = 0.142, adjusted R² = 0.101; residual SE = 6.584

Model: MD ~ FAZ Area + age + sex (n = 68)

Variable	β	SE	95% CI	p-value	Significance
Intercept	5.087	7.891	-10.676, 20.850	0.5214	n.s.
**FAZ Area**	-0.903	10.752	-22.383, 20.578	0.9334	n.s.
Age (years)	-0.143	0.119	-0.380, 0.094	0.2334	n.s.
Sex (female=1)	-3.273	1.698	-6.665, 0.120	0.0584	n.s.

Model fit: F = 1.79, p = 0.1573; R^2 = 0.078, adjusted R^2 = 0.034; residual SE = 6.825

G. sine tensio

Model: MD ~ RNFL avg + age + sex (n = 28)

Variable	β	SE	95% CI	p-value	Significance
Intercept	-10.977	11.987	-35.717, 13.762	0.3689	n.s.
**RNFL avg**	0.190	0.083	0.019, 0.362	0.0313	*
Age (years)	-0.185	0.129	-0.450, 0.080	0.1626	n.s.
Sex (female=1)	0.961	1.910	-2.980, 4.902	0.6194	n.s.

Model fit: F = 3.92, p = 0.0208; R^2 = 0.329, adjusted R^2 = 0.245; residual SE = 4.558

Model: MD ~ Avg GCC + age + sex (n = 28)

Variable	β	SE	95% CI	p-value	Significance
Intercept	-9.130	12.594	-35.123, 16.864	0.4755	n.s.
**Avg GCC**	0.182	0.094	-0.012, 0.376	0.0648	n.s.
Age (years)	-0.198	0.132	-0.469, 0.073	0.1453	n.s.
Sex (female=1)	2.649	1.810	-1.087, 6.385	0.1563	n.s.

Model fit: F = 3.31, p = 0.0371; R^2 = 0.293, adjusted R^2 = 0.204; residual SE = 4.678

Model: MD ~ VD Inner + age + sex (n = 28)

Variable	β	SE	95% CI	p-value	Significance
Intercept	-0.908	10.718	-23.029, 21.213	0.9332	n.s.
**VD Inner**	0.518	0.332	-0.167, 1.204	0.1318	n.s.
Age (years)	-0.248	0.131	-0.518, 0.023	0.0709	n.s.
Sex (female=1)	2.378	1.862	-1.465, 6.221	0.2138	n.s.

Model fit: F = 2.78, p = 0.0632; R^2 = 0.258, adjusted R^2 = 0.165; residual SE = 4.793

Model: MD ~ Perf Inner + age + sex (n = 28)

Variable	β	SE	95% CI	p-value	Significance

Intercept	-2.761	10.709	-24.864, 19.342	0.7987	n.s.
**Perf Inner**	35.732	19.773	-5.078, 76.542	0.0833	n.s.
Age (years)	-0.262	0.129	-0.528, 0.004	0.0531	n.s.
Sex (female=1)	2.576	1.826	-1.194, 6.346	0.1713	n.s.

Model fit: F = 3.11, p = 0.0450; R^2 = 0.280, adjusted R^2 = 0.190; residual SE = 4.719

Model: MD ~ FAZ Area + age + sex (n = 28)

Variable	β	SE	95% CI	p-value	Significance
Intercept	8.785	9.146	-10.091, 27.660	0.3464	n.s.
**FAZ Area**	-1.424	9.736	-21.519, 18.670	0.8849	n.s.
Age (years)	-0.249	0.149	-0.557, 0.058	0.1075	n.s.
Sex (female=1)	2.592	1.968	-1.471, 6.655	0.2003	n.s.

Model fit: F = 1.79, p = 0.1757; R^2 = 0.183, adjusted R^2 = 0.081; residual SE = 5.028

Full coefficients, standard errors, 95% confidence intervals, and p-values for each predictor variable (parameter of interest, age, sex), plus overall model statistics (F-statistic with p-value, R^2, adjusted R^2, residual standard error). Sex coded as female=1, male=0.

Table S4A. Kruskal–Wallis comparisons of macular OCTA and structural parameters across the three glaucoma subtypes: full cohort (n=197) versus one-eye-per-patient subset (n=191).

Variable	Full cohort p	One-eye p	Concordant?
Macular OCTA — Vessel Density			
VD Central	0.005**	0.005**	Yes (significant in both)
VD Inner	<0.001***	<0.001***	Yes (significant in both)
VD Full	<0.001***	<0.001***	Yes (significant in both)
Macular OCTA — Perfusion Density			
Perfusion Central	0.035*	0.045*	Yes (significant in both)
Perfusion Inner	<0.001***	<0.001***	Yes (significant in both)
Perfusion Full	<0.001***	<0.001***	Yes (significant in both)
FAZ Morphology			
FAZ Area	<0.001***	<0.001***	Yes (significant in both)
FAZ Perimeter	0.008**	0.009**	Yes (significant in both)
FAZ Circularity	<0.001***	<0.001***	Yes (significant in both)
Structural Parameters — GCC			
Avg GCC	0.052	0.068	Yes (ns in both)

Variable	Full cohort p	One-eye p	Concordant?
Min GCC	0.096	0.109	Yes (ns in both)
Structural Parameters — RNFL			
RNFL avg	0.037*	0.085	Borderline shift (significance lost in sensitivity)
RNFL Superior	0.015*	0.038*	Yes (significant in both)
RNFL Nasal	0.458	0.439	Yes (ns in both)
RNFL Inferior	0.286	0.439	Yes (ns in both)
RNFL Temporal	0.369	0.431	Yes (ns in both)
Other			
CST	0.874	0.876	Yes (ns in both)
MD (dB)	0.028*	0.053	Borderline shift
PSD (dB)	<0.001***	<0.001***	Yes (significant in both)

Kruskal–Wallis between-subtype comparisons across POAG, PXG, and NTG. * $p<0.05$; ** $p<0.01$; *** $p<0.001$; ns = not significant. All nine macular OCTA parameters preserve their significance status between the full and one-eye analyses; minor borderline shifts on RNFL avg and MD reflect loss of statistical power on a smaller subset rather than substantive change.

Table S4B. Bonferroni-corrected Mann–Whitney pairwise post hoc comparisons for the principal macular OCTA parameters: full cohort versus one-eye-per-patient subset.

Variable	Pair	Full p (Bonferroni)	One-eye p (Bonferroni)
VD Inner			
VD Inner	POAG vs. PXG	<0.001***	<0.001***
VD Inner	POAG vs. NTG	0.379	0.397
VD Inner	PXG vs. NTG	0.129	0.144
Perfusion Inner			
Perf Inner	POAG vs. PXG	<0.001***	<0.001***
Perf Inner	POAG vs. NTG	0.869	0.901
Perf Inner	PXG vs. NTG	0.043*	0.050*
FAZ Area			
FAZ Area	POAG vs. PXG	0.006**	0.005**
FAZ Area	POAG vs. NTG	0.209	0.231
FAZ Area	PXG vs. NTG	0.006**	0.006**
FAZ Circularity			
FAZ Circularity	POAG vs. PXG	<0.001***	<0.001***
FAZ Circularity	POAG vs. NTG	0.225	0.166
FAZ Circularity	PXG vs. NTG	0.102	0.077
Avg GCC			

Variable	Pair	Full p (Bonferroni)	One-eye p (Bonferroni)
Avg GCC	POAG vs. PXG	1.000	1.000
Avg GCC	POAG vs. NTG	0.163	0.124
Avg GCC	PXG vs. NTG	0.055	0.082

Mann–Whitney U pairwise post hoc comparisons after Kruskal–Wallis, Bonferroni-corrected for three pairwise contrasts. The pattern of significant subtype contrasts is preserved between the full cohort and the one-eye-per-patient subset for every key OCTA variable. * p<0.05; ** p<0.01; *** p<0.001.

Table S4C. 5-fold cross-validated ROC analysis: structural-only, OCTA-only, and combined logistic-regression models across the three pairwise subtype comparisons. Full cohort versus one-eye-per-patient subset.

Comparison	Model	Full AUC ± SD	One-eye AUC ± SD
POAG vs. PXG			
POAG vs. PXG	Structural-only	0.539 ± 0.155	0.515 ± 0.094
POAG vs. PXG	OCTA-only	0.715 ± 0.059	0.702 ± 0.103
POAG vs. PXG	Combined OCTA+Struct.	0.690 ± 0.043	0.677 ± 0.112
POAG vs. NTG			
POAG vs. NTG	Structural-only	0.520 ± 0.118	0.555 ± 0.173
POAG vs. NTG	OCTA-only	0.565 ± 0.122	0.549 ± 0.049
POAG vs. NTG	Combined OCTA+Struct.	0.673 ± 0.120	0.636 ± 0.150
PXG vs. NTG			
PXG vs. NTG	Structural-only	0.601 ± 0.196	0.627 ± 0.095
PXG vs. NTG	OCTA-only	0.763 ± 0.039	0.753 ± 0.058
PXG vs. NTG	Combined OCTA+Struct.	0.743 ± 0.132	0.781 ± 0.076

Logistic regression with L2 regularisation (C=0.1), 5-fold stratified cross-validation. Combination models include all 9 OCTA parameters (OCTA-only), all 8 structural parameters (Structural-only), or all 17 (Combined). The qualitative ranking of model performance — OCTA-only and Combined outperforming Structural-only for POAG vs. PXG and PXG vs. NTG; combined model providing the best discrimination for POAG vs. NTG — is preserved in the one-eye-per-patient subset.

Table S4D. Multivariate OLS regression of mean deviation (MD) on the principal OCTA and structural predictors within each subtype, adjusted for age and sex. Full cohort versus one-eye-per-patient subset.

Predictor	Subtype	Full β	Full p	One-eye β	One-eye p
Perfusion Inner					
Perf Inner	POAG	13.704	0.168	14.713	0.158
Perf Inner	PXG	−32.860	0.035*	−32.863	0.038*
Perf Inner	NTG	35.732	0.083	35.732	0.083
VD Inner					

Predictor	Subtype	Full β	Full p	One-eye β	One-eye p
VD Inner	POAG	0.222	0.160	0.235	0.155
VD Inner	PXG	−0.374	0.144	−0.374	0.152
VD Inner	NTG	0.518	0.132	0.518	0.132
RNFL avg					
RNFL avg	POAG	0.128	0.002**	0.125	0.003**
RNFL avg	PXG	0.156	0.003**	0.166	0.003**
RNFL avg	NTG	0.190	0.031*	0.190	0.031*
Avg GCC					
Avg GCC	POAG	0.082	0.064	0.078	0.089
Avg GCC	PXG	0.193	0.003**	0.203	0.003**
Avg GCC	NTG	0.182	0.065	0.182	0.065

Linear regression with MD (dB) as outcome, adjusted for age and sex. β = unstandardised regression coefficient. The principal regression findings of the manuscript — Perfusion Inner as a significant negative predictor of MD specifically in PXG; RNFL avg as a consistent positive predictor across subtypes; Avg GCC as significant in PXG — are reproduced with virtually identical coefficients and statistical significance in the one-eye-per-patient subset. * $p<0.05$; ** $p<0.01$.

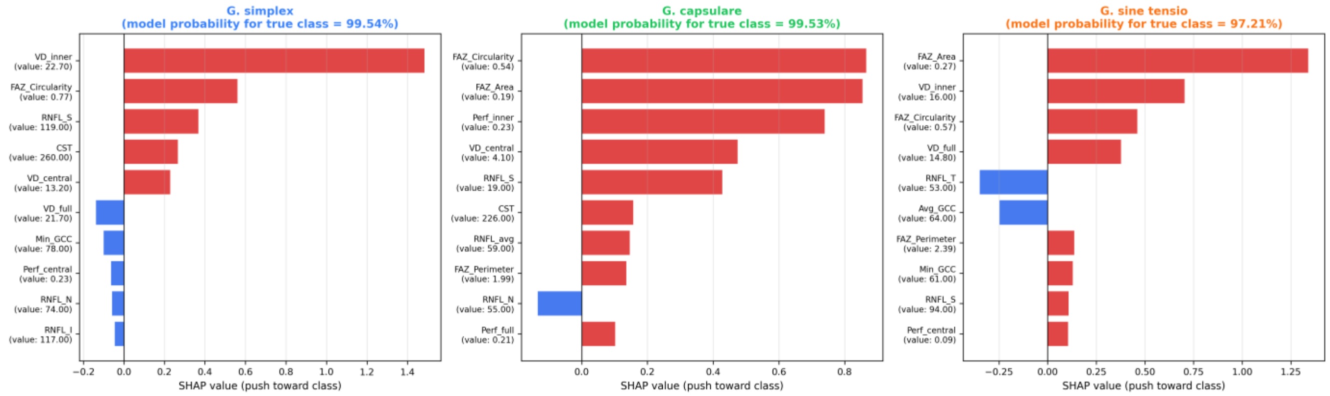

Figure S1. SHAP (SHapley Additive exPlanations) feature contribution plots for one representative case from each of the three glaucoma subtypes, selected as the example with the highest model probability for the correct subtype class. Each bar represents the contribution of a single OCT/OCTA parameter to the model's decision for that patient. Red bars indicate features that push the prediction toward the true class; blue bars indicate features that push the prediction away. Feature values are shown in parentheses. In G. simplex, VD Inner was the dominant positive contributor; in G. capsulare, FAZ Circularity, FAZ Area, and Perf Inner were the top contributors, reflecting the combined macular perfusion and geometric FAZ signature of pseudoexfoliative disease; in G. sine tensio, FAZ Area was the dominant positive contributor, consistent with enlarged foveal avascular zone as the hallmark of primary vascular dysregulation in NTG.

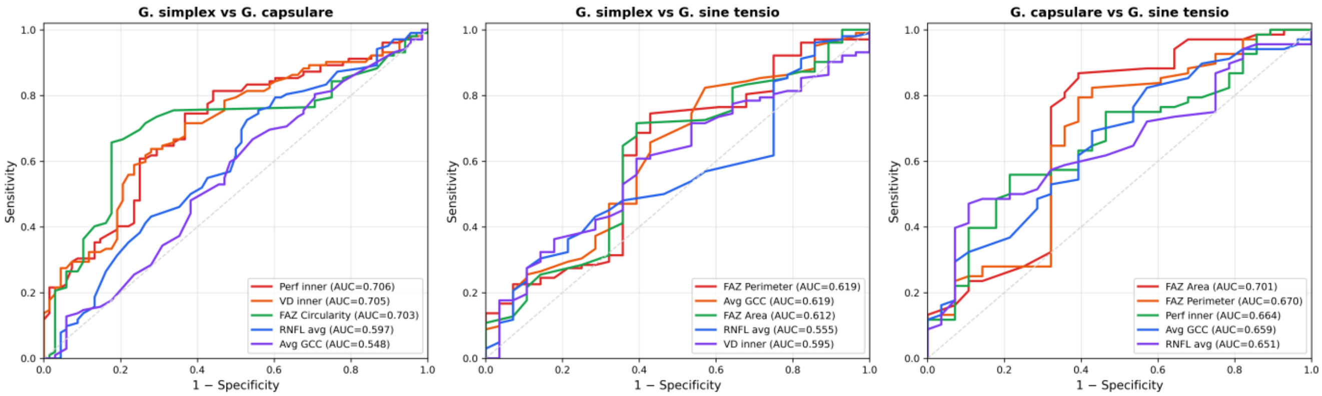

Figure S2. Receiver operating characteristic (ROC) curves for the top five individual parameters (OCTA and structural) in each of the three pairwise glaucoma subtype comparisons. AUC values are shown in the legend; dashed diagonal line represents chance discrimination (AUC = 0.5). (A) G. simplex vs. G. capsulare: Perf Inner (AUC=0.706), VD Inner (0.705), and FAZ Circularity (0.703) all achieved AUC>0.70, substantially outperforming structural parameters (RNFL avg=0.597, Avg GCC=0.548). (B) G. simplex vs. G. sine tensio: FAZ Perimeter (0.619), Avg GCC (0.619), and FAZ Area (0.612) performed comparably, with no single parameter achieving strong discrimination; this is consistent with the structurally similar phenotypes of POAG and NTG. (C) G. capsulare vs. G. sine tensio: FAZ Area (0.701) and FAZ Perimeter (0.670) emerged as the top discriminators, confirming FAZ morphology as the primary vascular signature that distinguishes pseudoexfoliative glaucoma from normal-tension glaucoma.
